# 3D cultured human medium spiny neurons functionally integrate and rescue motor deficits in Huntington’s disease mice

**DOI:** 10.1172/JCI187941

**Published:** 2025-10-15

**Authors:** Yuting Mei, Yuan Xu, Xinyue Zhang, Ban Feng, Yingying Zhou, Qian Cheng, Yuan Li, Xingsheng Peng, Mengnan Wu, Lianshun Xie, Lei Xiao, Wenhao Zhou, Yuejun Chen, Man Xiong

**Affiliations:** 1 State Key Laboratory of Brain Function and Disorders, MOE Frontiers Center for Brain Science, Institutes of Brain Science, Fudan University, Shanghai, China.; 2 Shanghai Key Laboratory of Birth Defects, Children’s Hospital of Fudan University, Shanghai, China.; 3 Institute of Neuroscience, Key Laboratory of Neuroscience, CAS Center for Excellence in Brain Science and Intelligence Technology, Chinese Academy of Sciences, Shanghai, China.; 4 University of Chinese Academy of Sciences, Beijing, China.

**Keywords:** Cell biology, Stem cells, Transplantation, Embryonic stem cells, Neurodegeneration, Stem cell transplantation

## Abstract

Dysfunction of striatal medium spiny neurons (MSNs) is implicated in several neurological disorders, including Huntington’s disease (HD). Despite progress in characterizing MSN pathology in HD, mechanisms underlying MSN susceptibility remain unknown, driving the need for MSNs derived from human pluripotent stem cells (hPSCs), especially subtypes in research and therapy. Here, we established a scalable 3D-default culture system to produce striatal MSNs efficiently from hPSCs by activation of the endogenous sonic hedgehog (SHH) pathway. These cells expressed canonical markers of striatal progenitors and dopamine D1 (D1) and dopamine D2 (D2) MSNs and presented dynamic specification and transcriptional signatures that closely resemble endogenous MSNs at single-cell resolution, both in vitro and post-transplantation in HD mice with quinolinic acid (QA) lesions. Grafted human cells survived and matured into D1-/D2-like MSNs and projected axons to endogenous targets including globus pallidus externus, globus pallidus internus, and substantia nigra pars reticulata to reconstruct the basal ganglia pathways. Functionally, they displayed spontaneous synaptic currents, received regulation from host cortex and thalamus, and were modulated by dopamine to either enhance or reduce neuronal excitability, similar to the endogenous D1-/D2-MSNs, subsequently improving behavior in QA-lesioned HD mice. Our study presents a method for generating authentic MSNs, providing a reliable cell source for HD cell therapy, mechanistic studies, and drug screening.

## Abstract 2

3D-default neural differentiation generates authentic human MSNs that funtionally integrate into host neural circuits and alleviate motor deficits in Huntington’s disease mice

## Graphical abstract

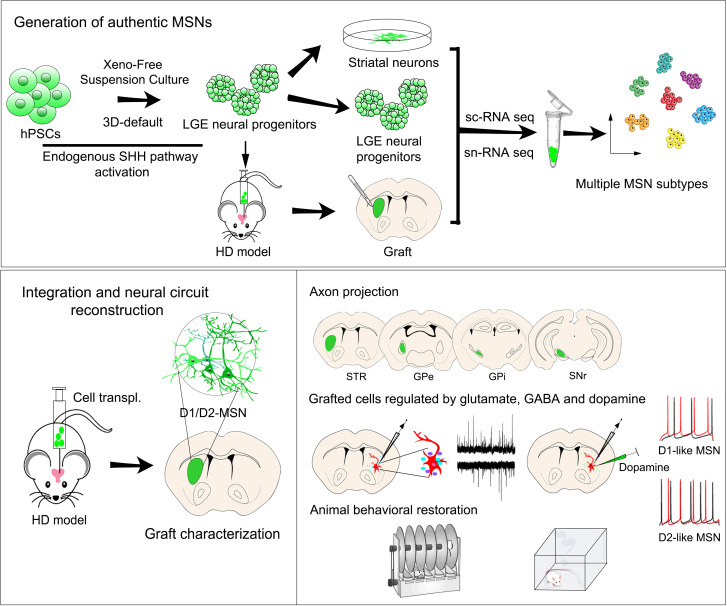

## Introduction

The striatum, the largest component and only input-receiving nucleus of the basal ganglia, is critically involved in control over conscious motor movements, executive functions, reward, and aversion (1). A large proportion of neurons in the striatum are MSNs, traditionally categorized into dopamine D1 (D1) and dopamine 2 (D2) subtypes based on distinct anatomic, molecular, and electrophysiological characteristics. The D1-MSN subtype expresses markers such as D1 receptor and substance P (SP) and projects mainly to the substantia nigra pars reticulata (SNr) and globus pallidus internus (GPi) to form the direct pathway. In contrast, the D2-MSN subtype is characterized by the expression of D2 receptor and enkephalin (ENK), with projections primarily targeting the globus pallidus externus (GPe) and, subsequently, the subthalamic nucleus, constituting the indirect pathway (2). Functionally, the direct pathway facilitates movement initiation and locomotion, whereas the indirect pathway suppresses movement initiation and terminates ongoing locomotion (3). Dysfunction of striatal MSNs in many neurodegeneration disorders, including HD and Parkinson’s disease (PD) and developmental disorders such as autism spectrum disorders, has been evidenced by studies using animal models and postmortem brains (4, 5). Although striatal neurodegeneration and MSN pathology are well characterized in HD animal models, the mechanisms underlying the selective vulnerability of D1- and D2-MSNs remain unclear (6).

The differentiation of hPSCs into striatal MSNs, especially MSN subtypes that closely resemble those present in the human striatum, holds promise for investigating the underlying pathogenic mechanisms and developing novel treatments (7). Normally, generation of striatal neurons from hPSCs in vitro is based on the same principle that takes place in the lateral ganglionic eminence (LGE) in vivo (8). Various protocols to produce striatal MSNs have been established, either by modulation of the sonic hedgehog (SHH) and/or WNT signaling pathways (9–12), or using activin A, which is an activator of the TGF-β pathway (13), to obtain a substantial proportion of MSNs characterized by dopamine- and cAMP-regulated phosphoprotein, molecular weight 32 kDa (DARPP32) or GABA expression in combination with COUP-TF–interacting protein 2 (CTIP2). We have previously differentiated MSNs from human embryonic stem cells (hESCs) that reconstruct basal ganglia neural circuits in hypoxic-ischemic injured brain by an embryoid body (EB) method (12). However, generating authentic MSNs, particularly MSN subtypes from hPSCs in vitro, remains challenging, impeding subtype-based studies and therapies for HD (14).

The pivotal requirement for successful stem cell therapies is that the transplanted neural progenitors should develop and mature into the appropriate neuron types or subtypes in terms of transcriptomics, morphology, and electrophysiological properties, and must also integrate into the existing neural circuits of the host brain (15). The capacity of grafted neural progenitors to extend axons and reconstruct host neural circuits largely depends on the cell fate determined in vitro and the intrinsic transcriptional programs that guide their subsequent differentiation into specific neuronal subtypes in vivo (16, 17). Therefore, it is crucial to differentiate MSNs in vitro with an expression profile that faithfully resembles that of the developing human striatum. These include key transcription factors governing LGE neural progenitor generation (FOXP1, DLX1/2, GSH1/2, MEIS2, GSX) (18, 19), and striatal MSN subtype specification/maturation (FOXP2, EBF1, ISL1, SP9, SIX3) (20, 21). Nonetheless, a comprehensive single-cell transcriptomic characterization of striatal neural progenitors and neurons derived from hPSCs in vitro is limited (22), and their eventual cell fate post-transplantation in vivo remains elusive. In addition, MSNs receive 2 main extrinsic inputs: glutamatergic afferents from cortical, limbic, and thalamic areas and dopaminergic afferents from the mesencephalon (23). However, it remains unclear whether the grafted MSNs in HD mice can functionally integrate into these neural circuits and respond to dopamine (DA), a key neuromodulator targeting D1/D2 receptors on striatal MSNs.

In this study, we developed a 3D suspension culture system that is free of animal-derived components and ventralizing morphogens, allowing for the generation of MSNs and their subtypes efficiently in vitro. The transcriptomic characteristics of differentiated cells show high similarities with the endogenous fetal brain at single-cell resolution in vitro and in vivo. After transplantation, the grafted neural progenitors matured into D1-/D2-MSNs, integrated into the host basal ganglia neural circuitry anatomically and functionally, and rescued animal behavior in the long term. Our research provides an effective approach to differentiate striatal D1- and D2-MSNs from hPSCs, which could be used to explore the underlying mechanisms of and advance stem cell therapy for MSN-related neurological disorders.

## Results

### 3D-default xeno-free suspension culture generates LGE progenitors efficiently through activation of the endogenous SHH signaling pathway.

**Figure 1:**
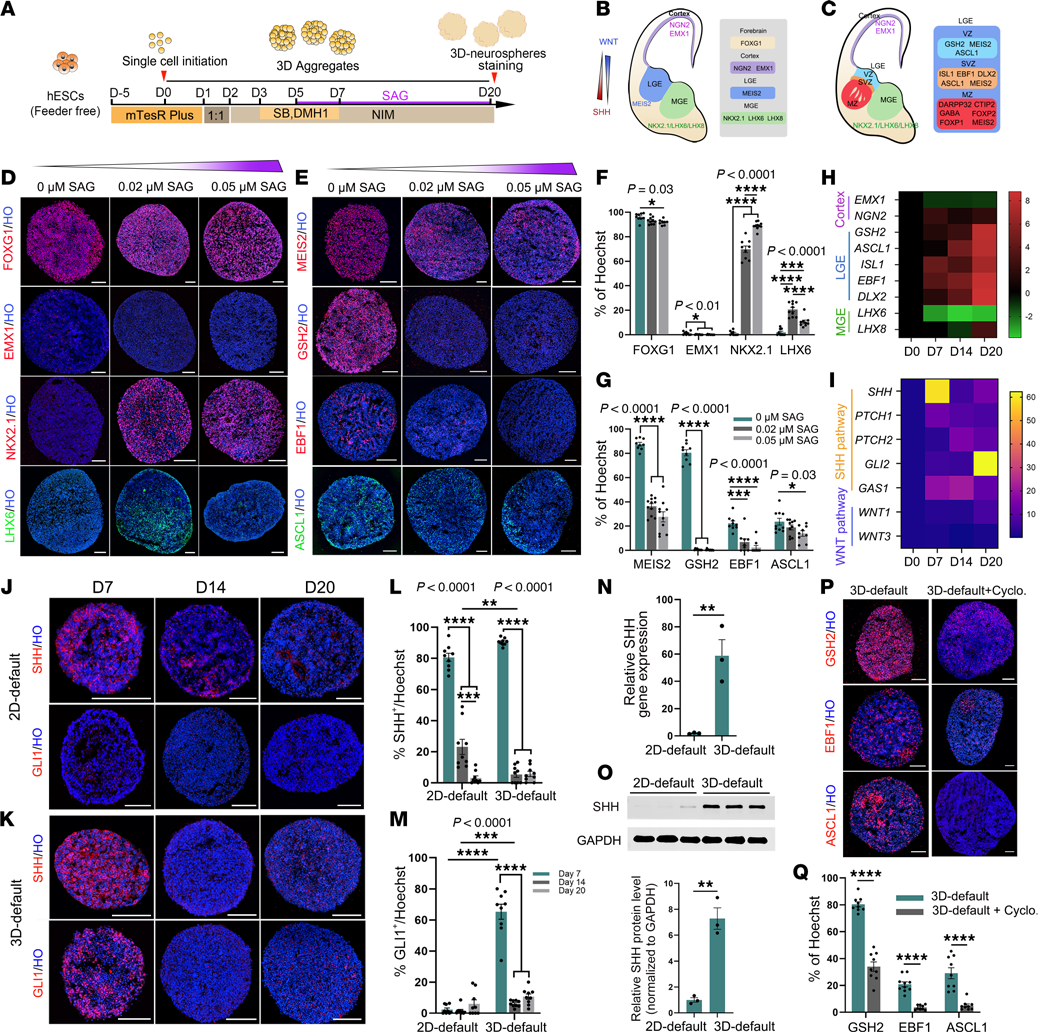
Generation and characterization of LGE progenitors derived from hESCs. (**A**) Schematic diagram illustrating timings and factors used for differentiating LGE-like progenitors from hESCs. (**B** and **C**) Schematic diagrams of signaling pathways and transcription factors involved in the development of different brain regions during forebrain development. (**D** and **F**) Representative immunofluorescence images (**D**)and quantification (**F**) for the brain region markers FOXG1, EMX1, NKX2.1, and LHX6 in day 20 neurospheres. Scale bar: 100 μm. (**E** and **G**) Representative immunofluorescence images (**E**) and quantification (**G**) for the expression of transcriptional factors including MEIS2, GSH2, EBF1, and ASCL1 in day 20 neurospheres. Scale bar: 100 μm. (**H**) Gene expression of representative brain region–specific markers at different times during neural differentiation from 3D-default XFSC. Color scale shows log2 fold change compared with day 0. (**I**) Relative mRNA expression levels of SHH and WNT signaling pathway–related genes in neurospheres derived from 3D-default XFSC on days 7, 14, and 20. Color scale shows relative expression level. (**J** and **K**) Representative immunofluorescence images of SHH and GLI1 protein expression in neurospheres from 2D-default culture (**J**) and 3D-default XFSC (**K**) at days 7, 14, and 20 during neural differentiation. Scale bar: 100 μm. (**L** and **M**) Quantifications of SHH^+^ and GLI1^+^ cells to total cells at different stages during neural differentiation. (**N** and **O**) qRT-PCR and Western blot analysis of SHH gene and protein expression in day 7 neurospheres differentiated by 2D-default and 3D-default culture. (**P** and **Q**) Representative immunofluorescence images (**P**) and quantification (**Q**) of GSH2^+^, EBF1^+^, and ASCL1^+^ cells in day 20 neurospheres generated by 3D-default XFSC, with or without Cyclo treatment. Scale bar: 100 μm. Data were analyzed by Student’s *t* test (for 2-group comparisons) or 1-way ANOVA (for multiple groups) and presented as the mean ± SEM; *n* = 3 biological replicates. *P < 0.05, **P < 0.01, ***P < 0.001, ****P < 0.0001.

To explore an approach that is suitable for large-scale therapeutic applications, we test in this study a neural differentiation strategy named 3D-XFSC (xeno-free suspension culture) to generate striatal neural progenitors (Figure 1A). Given that WNT and SHH signaling dorsoventrally regulate the patterning of LGE-derived striatal neural progenitors (Figure 1B), we hypothesize that appropriate activation of the SHH pathway would enhance LGE progenitor production. Thus, we treated 3D-XFSC–derived spheres with smoothened agonist (SAG), the agonist of SHH pathway, with different doses (0.00, 0.02, and 0.05 μM) from day 5 to day 20 during neural differentiation. Immunostaining and statistical analysis revealed that greater than 95% of the cells expressed forebrain marker forkhead box G1 (FOXG1) and less than 5% expressed cortical progenitor marker empty spiracles homeobox 1 (EMX1) in day 20 neurospheres across all 3 doses of SAG (Figure 1, D and F).

Surprisingly, we found 87.56% ± 1.53% of the cells were stained by homeobox protein meis2 (MEIS2), an LGE marker, in the 0 μM SAG condition. In contrast, only 36.39% ± 2.14% and 27.70% ± 4.18% of the cells were MEIS2^+^ in the 0.02 and 0.05 μM SAG groups, respectively (Figure 1, E and G). For MGE-derived progenitors, although there were 69.8% ± 2.68 % and 88.95% ± 0.96% NK2 homeobox 1–positive (NKX2.1^+^) cells, and 20.73% ± 1.76% and 10.61% ± 1.38% LHX6^+^ cells with SAG treatment (0.02 μM and 0.05 μM), respectively, less than 3% of total cells were stained by NKX2.1 or LHX6 without SAG treatment (Figure 1, D and F).

During striatal development, early LGE neural progenitors differentiate into distinct subtypes in the ventricular, subventricular, and the mantle zones with expression of various transcription factors (Figure 1C) (24). To investigate if LGE neural progenitors differentiated by 3D-XFSC could further develop into their subtypes, transcriptional factors GSH2, ASCL1, and EBF1 for the LGE ventricular zone and/or subventricular zone were examined by immunostaining (Figure 1E). The statistical analysis revealed that 80.53% ± 2.5 %, 22.3% ± 1.98%, and 23.70% ± 2.78% of the total cells expressed GSH2, EBF1, and ASCL1, and SAG treatment significantly reduced their expression (Figure 1G). Furthermore, quantitative real-time PCR (qRT-PCR) data revealed progressive upregulation of striatal progenitor markers (GSH2, ASCL1, ISL1, EBF1, DLX2) from day 0 to day 20 during in vitro differentiation. In contrast, dorsal telencephalic (EMX1, NGN2) and MGE (LHX6, LHX8) markers remained minimally expressed throughout the entire differentiation process (Figure 1H). These findings indicate striatal neural progenitors can be achieved efficiently without the use of SAG, and we named this method 3D-default XFSC in this study.

Given the in vivo evidence demonstrating that SHH signaling is crucial for LGE development (25), we hypothesize that 3D-default XFSC induces striatal neural progenitors by activating the endogenous SHH signaling pathway. We performed qRT-PCR to assess the expression of genes related to SHH and WNT signaling pathways throughout neural differentiation. The gene heatmap revealed gradual upregulation of the following SHH pathway components: ligand (SHH), receptor (PTCH1, PTCH2), coreceptor (GAS1), and downstream effector (GLI2). However, genes associated with the WNT pathway, such as WNT1 and WNT3, crucial for dorsal forebrain patterning (26), exhibited sustained low expression across day 0 to day 20 (Figure 1I).

To further confirm activation of endogenous SHH pathway in neurospheres from 3D-default XFSC, we used neural progenitors with cortical identity (Supplemental Figure 1, H–L; supplemental material available online with this article; https://doi.org/10.1172/JCI187941DS1), differentiated by the 2D-default EB (2D-default) culture (27), as a control. We evaluated the gene and protein expressions of SHH, along with the protein level of GLI1, a key downstream effector of SHH signaling. Surprisingly, SHH protein was detected in both 2D-default and 3D-default XFSC neurospheres at day 7, then expression gradually decreased from day 14 to day 20 during differentiation (Figure 1, J–L). Notably, while SHH was apparent in 2D-default–differentiated spheres at day 7, the expression was diffuse and weak, in contrast to strong membrane-localized SHH in 3D-default XFSC (Figure 1, J and K). To further determine SHH expression levels between these 2 methods, we performed qRT-PCR and Western blot analysis. The results showed that both SHH mRNA and protein were highly expressed in 3D-default XFSC compared with that of 2D-default culture (Figure 1, N and O). Importantly, the protein level of GLI1, was notably higher in neurospheres from 3D-default XFSC compared with those from the 2D-default at day 7, followed by a significant decrease from day 14 to day 20 during neural differentiation (Figure 1, K and M).

To investigate when the SHH pathway activates and critically promotes LGE features, we collected spheres generated by 3D-default-XFSC every 2 days from day 0 to day 17 during neural differentiation, and we dynamically explored the expression of SHH and GLI1, as well as striatal progenitor markers GSH2 and MEIS2 (Supplemental Figure 1, A–C). Statistical analysis revealed that SHH expression began at day 5, peaked at day 9, and decreased from day 11, whereas GLI1 expression occurred from day 7 and persisted until day 11. Subsequently, MEIS2 began to be expressed at day 11 and persisted at a high level through day 17, followed by GSH2 expression from day 17 (Supplemental Figure 1, D–G). These results indicate a correlation between SHH signaling pathway activation and LGE fate acquisition during 3D-default XFSC neural differentiation.

Finally, to determine whether inhibition of the endogenous SHH pathway would affect generation of LGE progenitors, neurospheres from 3D-default XFSC were treated with or without cyclopamine (Cyclo), an inhibitor of the SHH signaling pathway. The immunostaining and statistical analyses showed that Cyclo treatment dramatically decreased cell proportions of GSH2, EBF1, and ASCL1 to total cells (Figure 1, P and Q).

In conclusion, these findings demonstrate that 3D-default XFSC generates LGE neural progenitors successfully and effectively by activation of the endogenous SHH signaling pathway.

### 3D-default XFSC produces D1- and D2-MSNs from hPSCs in vitro.

**Figure 2:**
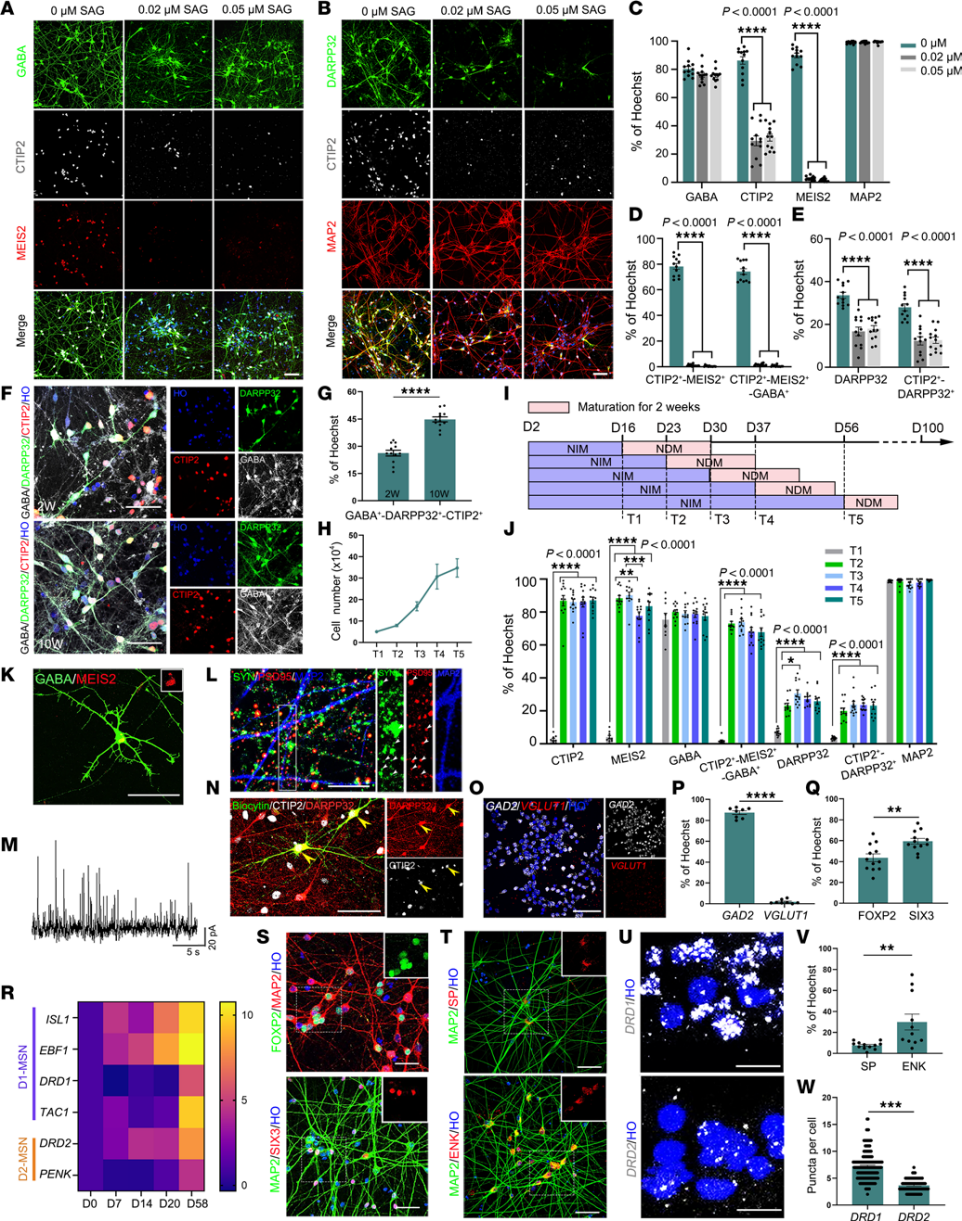
Generation and characterization of striatal MSN subtypes from hESCs by 3D-default XFSC. (**A** and **B**) Immunostaining of CTIP2/MEIS2/GABA/HO and CTIP2/MAP2/DARPP32/HO in neurons generated by 3D-default XFSC with or without SAG 2 weeks after neuron maturation. Scale bar: 50 μm. (**C**–**E**) Quantification of cells positive for GABA, CTIP2, MEIS2, MAP2, DARPP32, or marker combinations (CTIP2/MEIS2, CTIP2/MEIS2/GABA, DARPP32/CTIP2) among total cells with or without SAG treatment. (**F** and **G**) Representative images (**F**) and quantification (**G**) of GABA^+^/DARPP32^+^/CTIP2^+^ cells among total cells after neuron maturation for 2 and 10 weeks. Scale bar: 50 μm. (**H**) Growth curve showing long-term expansion capacity of 3D-default XFSC–derived LGE-like progenitors. (**I**) Schematic diagram illustrating timings of neural differentiation and maturation with 3D-default XFSC. (**J**) Quantification of cells positive for GABA, CTIP2, MEIS2, DARPP32, MAP2, or marker combinations (CTIP2/MEIS2/GABA, DARPP32/CTIP2) among total neurons across different patterning time points (T1–T5) with 3D-default XFSC. (**K**) Immunofluorescence image showing a GABA/MEIS2 double-positive neuron with typical spiny morphology. Scale bar: 50 μm. (**L**) Postsynaptic density protein 95 (PSD95), MAP2, and SYN immunostaining showing prominent co-localization of PSD95 and SYN. Scale bar: 10 μm. (**M** and **N**) Typical trace of sIPSCs (**M**) and post-recording immunostaining images (**N**) for biocytin/DARPP32/CTIP2. Scale bar: 50 μm. (**O** and **P**) RNAscope staining (**O**) and quantification (**P**) of *GAD2* and *VGLUT1*. Scale bar: 50 μm. (**R**) Relative mRNA expression levels of canonical genes for D1- and D2-MSNs at different stages during 3D-default XFSC. (**Q**, **S**, **T**, and **V**) Representative immunofluorescence images (**S** and **T**) and quantification (**Q** and **V**) of FOXP2 and SP for D1-MSN, and SIX3 and ENK for D2-MSN. Scale bar: 50 μm. (**U** and **W**) RNAscope staining (**U**) and quantification (**W**) of *DRD1* and *DRD2*. Scale bar: 50 μm. Data were analyzed by Student’s *t* test (for 2-group comparisons) or 1-way ANOVA (for multiple groups) and presented as the mean ± SEM; *n* = 3 biological replicates. *P < 0.05, **P < 0.01, ***P < 0.001, ****P < 0.0001.

To investigate whether LGE neural progenitors generated by 3D-default-XFSC could further differentiate into mature MSNs and their subtypes, hESC-derived neural spheres treated with different doses of SAG were dissociated and matured on glass coverslips at 30 days after neural differentiation (Supplemental Figure 2A). Neurons were examined 2 weeks after maturation by immunostaining with GABA/MEIS2/CTIP2/HO and MAP2/DARPP32/CTIP2/HO (Figure 2, A and B). Interestingly, greater than 95% of the cells expressed the neuronal marker MAP2, and approximately 80% of cells were GABAergic neurons (GABA^+^) across all 3 dose groups (0.00, 0.02, and 0.05 μM SAG) without significant deviation (Figure 2C). These data indicate an exceptionally high efficiency of GABAergic neuron differentiation via 3D-XFSC culture. Notably, CTIP2, a crucial transcription factor in MSN differentiation, was expressed in nearly 90% of untreated cells but declined to less than 40% after treatment with 0.02 or 0.05 μM SAG. Moreover, the striatal transcription factor MEIS2 was dramatically reduced from 89.92% ± 1.63% (0 μM SAG) to less than 3% in SAG-treated cells (Figure 2C). We also observed that 78.22% ± 2.21% cells were double-stained by MEIS2 and CTIP2, and 74.2% ± 2.21% cells were triple-labeled by GABA, MEIS2, and CTIP2 in the 0.00 μM SAG group, whereas the groups treated with 0.02 or 0.05 μM SAG exhibited significant reduction (Figure 2D).

Finally, the proportion of DARPP32^+^ cells, either alone or colabeled with CTIP2, was significantly higher in the 0.00 μM SAG group (33.65% ± 1.42% and 28.05% ± 1.62%, respectively) than 0.02 μM (16.77% ± 2.03% and 12.38% ± 1.89%, respectively) or 0.05 μM (18.04% ± 1.42% and 12.69% ± 1.46%, respectively) SAG (Figure 2E). More importantly, to investigate the production of fully mature MSNs by 3D-default-XFSC, we stained GABA/DARPP32/CTIP2 using tyramide signal amplification technology at 2 and 10 weeks after neuron maturation. The proportion of GABA^+^/DARPP32^+^/CTIP2^+^ cells increased significantly from 26.57% ± 1.58% at week 2 to 44.84% ± 1.38% by 10 weeks of maturation (Figure 2, F and G). We also performed triple labeling of GAD67 (an alternative GABAergic marker) with CTIP2 and DARPP32, following Conforti et al. (22). The results showed increased co-expression from 27.51% ± 2.30% at 2 weeks to 40.14% ± 1.85% at 10 weeks after maturation (Supplemental Figure 2E). Notably, greater than 90% of DARPP32^+^-CTIP2^+^ cells were GAD67^+^ across all groups (Supplemental Figure 2F), confirming striatal GABAergic neurons. Trypan blue and TUNEL staining revealed that the viability of neurons after 10 weeks of in vitro culture was greater than 80% (Supplemental Figure 2, H–K). These results demonstrate that extended maturation is essential for striatal MSN development.

Interestingly, although SAG treatment significantly reduced the percentage of striatal neurons (GAD67^+^/CTIP2^+^/DARPP32^+^), it increased the percentage of medial ganglionic eminence-derived (MGE-derived) neurons (NKX2.1^+^/GABA^+^/CTIP2^−^) 2 weeks after neuron maturation in vitro (Supplemental Figure 2, B–D, and G), suggesting the acquisition of an MGE GABAergic neuronal fate. In addition, very few cells (<3%) expressed markers associated with cortical glutamatergic neurons (SATB2, CUX1, TBR1), cholinergic neurons (CHAT), dopaminergic neurons (TH), oligodendrocytes (MBP), astrocytes (GFAP), and interneurons (parvalbumin, calretinin) by 3D-default XFSC in vitro (Supplemental Figure 3, A and B). Moreover, human-induced pluripotent stem cells (iPSCs) showed comparable levels of LGE neural progenitors and striatal neurons generated by 3D-default XFSC (Supplemental Figure 3, C–F). Collectively, these findings indicate that hPSCs can be efficiently differentiated into striatal MSNs by 3D-default XFSC.

For cellular analysis or clinical application, it is crucial to maintain and expand the striatal neural progenitors without losing their ability to produce MSNs. To evaluate the expansion capacity and the cell fate after long-term maintenance in vitro, LGE neural progenitors at various stages (T1–T5) of neural differentiation were dissociated and cultured on coverslips for a 2-week maturation before immunostaining (Figure 2I). The statistical analyses showed that the neural progenitor cell numbers progressively increased by nearly 7-fold at T5 (34.75 ± 4.28 × 10^4^) compared with T1 (5.00 ± 0.00 × 10^4^), the first time point for cell harvesting (Figure 2H). More importantly, immunostaining and statistical analysis of MSNs markers (GABA, MEIS2, CTIP2, DARPP32, MAP2) revealed that neural progenitors from various stages consistently generated a substantial number of striatal MSNs stained by GABA^+^-CTIP2^+^-MEIS2^+^, and DARPP32^+^, as well as CTIP2^+^-DARPP32^+^, with no significant differences among these groups, except for neural progenitors from day 16 (Figure 2J). Of note, greater than 80% of the cells were positive for CTIP2 or MEIS2 across all time points except day 16, suggesting that day 16 to day 23 was important for the fate determination toward striatal identity. In addition, greater than 95%, or close to 80%, of total cells were labeled with MAP2 or GABA across all groups (Figure 2J), illustrating efficient generation of GABAergic neurons for 3D-default XFSC protocol in neurospheres from day 16 to day 56. In addition, when initiated with equal cell numbers, 3D-default XFSC generated significantly more neural progenitors than 2D-culture for MSNs (28) (Supplemental Figure 2, M and N). Overall, these findings indicate the superior efficiency and scalability of LGE neural progenitors differentiated by 3D-default XFSC, even in prolonged culture.

Next, we assessed whether MSN differentiated by 3D-default XFSC could further mature into functional MSNs and their subtypes in vitro. First, we observed that after 4 weeks maturation in vitro, differentiated human striatal MSNs developed spiny dendrites that closely resembled those of native MSNs(Figure 2K). The neurons co-expressed presynaptic SYN and postsynaptic density protein 95, suggesting the formation of synaptic connections between neurons in vitro (Figure 2L). Second, RNAscope staining revealed that 87.34% ± 1.73% of the cells expressed glutamic acid decarboxylase 2 (*GAD2*), suggesting synthesis of γ-aminobutyric acid neurotransmitter in these neurons (Figure 2, O and P). The whole-cell patch-clamp recordings revealed that all examined cells fired multiple action potentials (APs) in response to depolarizing current injections (Supplemental Figure 3, I and J). Voltage-clamp recordings also captured spontaneous inhibitory postsynaptic currents (sIPSCs) (Figure 2M) with amplitude of 19.79 ± 1.15 pA and frequency of 1.64 ± 0.37 Hz (Supplemental Figure 3, K and L), suggesting release of GABA neurotransmitters by these neurons. The single AP revealed electrophysiological properties, including resting membrane potential (RMP), threshold, and peak amplitude (Supplemental Figure 3, G and H). The recorded neurons were stained by DARPP32, biocytin, and CTIP2, suggesting striatal MSN identity (Figure 2N). These data indicated functional activity of striatal MSNs generated by 3D-default XFSC.

To determine MSN subtypes, we analyzed D1-MSN (ISL1, EBF1, DRD1, TAC1) and D2-MSN (DRD2, PENK) gene expression by qRT-PCR. The heatmap showed progressive upregulation of these subtype-specific genes in LGE progenitors from day 7 to day 58 (Figure 2R). Interestingly, DRD2 mRNA was detectable in day 14 neural progenitors, though at significantly lower levels than in day 58 neurons (Supplemental Figure 2L), suggesting potential roles in striatal progenitor development (29). Importantly, we noted a substantial population of cells expressing FOXP2 and SP for D1-MSN, as well as SIX3 and ENK for D2-MSN (Figure 2, Q, S, T, and V). Finally, RNAscope staining of *DRD1* and *DRD2* revealed a higher density of *DRD1*^+^ puncta compared with *DRD2*^+^ puncta 10 weeks after neuron maturation (Figure 2, U and W).

Together, these findings demonstrate efficient and scalable differentiation of human striatal neural progenitors into mature MSNs, including D1 and D2 subtypes, by 3D-default XFSC in vitro.

### Human striatal derivatives generated by 3D-default XFSC exhibit typical features of LGE progenitors and MSN subtypes at single-cell resolution.

**Figure 3:**
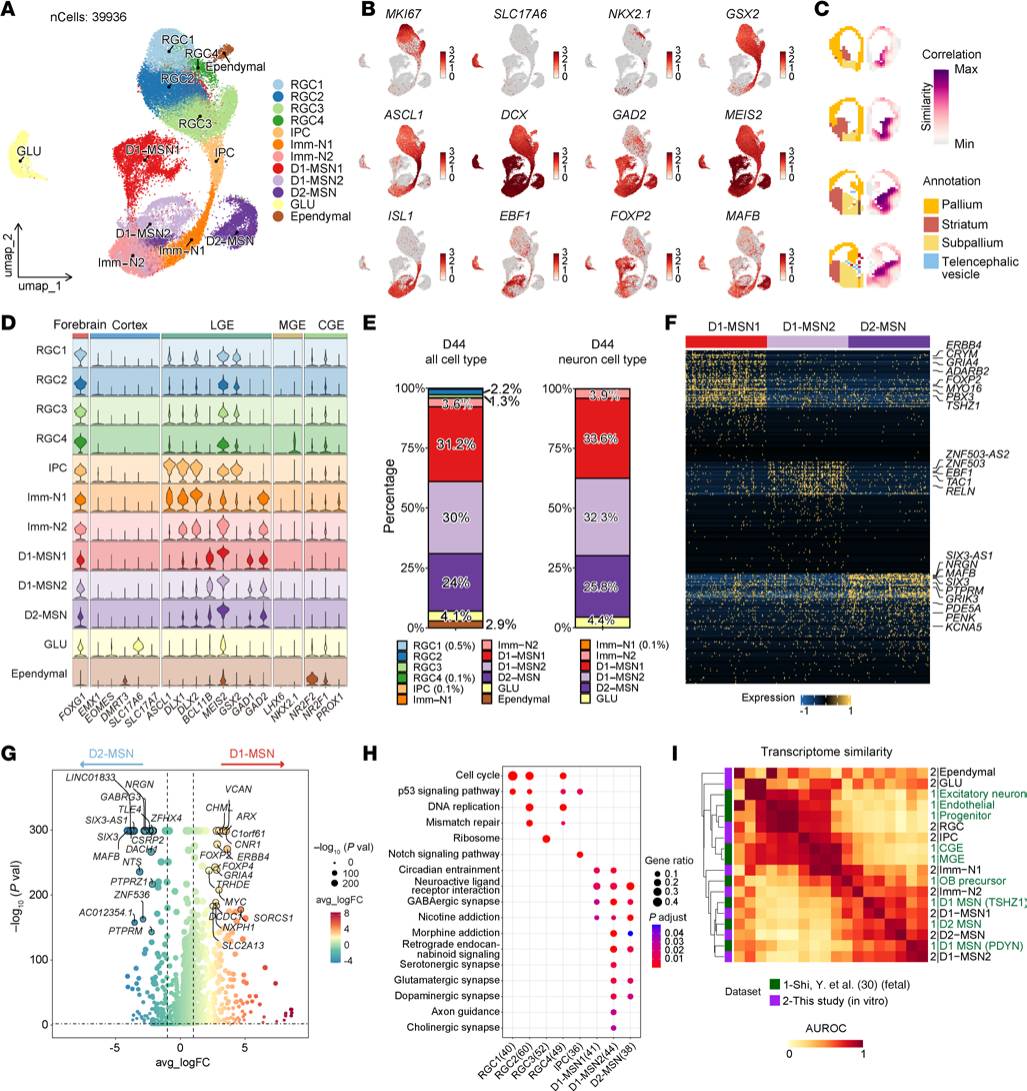
Specification and characterization of human LGE neural progenitor and MSN subtypes in vitro. (**A**) UMAP visualization of cell clusters identified from integrated scRNA-Seq data sets of day 20 and day 44 samples (*n* = 2 biological replicates per time point). (**B**) UMAP visualization the expression of marker genes for different cell types. (**C**) Spatial mapping of all cell clusters onto the E13 mouse brain using VoxHunt with Allen Developing Mouse Brain Atlas reference data. (**D**) Violin plots showing the expression of regional marker genes for the forebrain, cortex, LGE, MGE, and CGE. (**E**) Bar plots quantifying the proportions of cell types among total cells (left) and total neurons (right) in the day 44 data set. (**F**) Heatmap of the top 100 (DEGs) distinguishing MSN subtypes. (**G**) Volcano plot highlighting DEGs potentially driving the lineages divergence of D1-MSNs versus D2-MSNs (**H**) KEGG pathway enrichment analysis of DEGs across distinct cell clusters. (**I**) Heatmaps depicting pairwise transcriptional correlations of 3D-default XFSC–derived LGE progenitors and MSNs in vitro with cell types in the developing human brain data set (30). AUROC, area under the receiver operating characteristic curve; GLU, glutamatergic neuron; max, maximum; min, minimum.

To further characterize LGE-like progenitors and MSNs differentiated via 3D-default XFSC, we conducted scRNA-Seq at 2 developmental time points: day 20 (progenitor stage) and day 44 (neuron stage) (Supplemental Figure 4A). After filtering out low-quality cells, a total of 24,430 cells from day 20 and 15,506 cells from day 44 were obtained and analyzed collectively (Supplemental Figure 4, B–D). Unsupervised clustering of cellular transcriptional identities by uniform manifold approximation and projection (UMAP) identified 12 distinct cell clusters (Figure 3A and Supplemental Table 1), according to well-known cell type–specific marker genes (Figure 3B and Supplemental Figure 4E). Notably, we found *MEIS2* (LGE-lineage cells) and *FOXP1* (general MSNs) expressed in nearly all cell clusters. The LGE_intermediate progenitor cell (LGE_IPC) was mainly characterized by the expression of IPC marker gene *ASCL1* and striatal D1/D2 MSN fate determination genes *DLX1* and *ISL1* (Figure 3B and Supplemental Figure 4E). UMAP and the gene heatmap (Figure 3B and Supplemental Figure 4, E and G) revealed that D1-MSNs highly expressed characteristic genes such as *EBF1*, *TAC1*, *FOXP2*, *ZNF503*, and *DRD1*, whereas D2-MSNs highly expressed *MAFB*, *SIX3*, *PENK*, and *DRD2*. Critically, we identified molecularly distinct D1-MSN subtypes based on differential marker gene expression (Supplemental Figure 4, E and G), demonstrating that in vitro–differentiated striatal progenitors can generate heterogeneous D1-MSN populations. Notably, only radial glia cell 4 (RGC4) exhibited *NKX2.1* expression characteristic of MGE lineage identity (Figure 3, A and B).

Using the E13 Allen Developing Mouse Brain Atlas data set as a reference, VoxHunt mapping demonstrated a pronounced correlation between 3D-default XFSC–derived cells and the corresponding tissues from the mouse ganglionic eminence in vivo (Figure 3C).The violin plots in Figure 3D show that most cells highly expressed LGE neuronal marker genes (*ASCL1*, *DLX1*, *DLX2*, *BCL11B*, *MEIS2*, *GSX2*) and GABA-synthesizing enzyme genes (*GAD1* and *GAD2*), whereas very few cells expressed cortex-specific genes (*EMX1*, *EOMES*, *DMRT3, SLC17A6*, *SLC17A7*) and MGE linage genes (*LHX6*, *NKX2-1*). These findings suggest that 3D-default XFSC mainly produced cells with LGE identity. Interestingly, we detected substantial expression of caudal ganglionic eminence (CGE) markers (*NR2F2*, *NR2F1*, *PROX1*) in RGC1–RGC4 and Imm-N1/N2 populations (Figure 3D and Supplemental Figure 4F), suggesting a small number of intermingled CGE cells in these cell clusters. However, they are undistinguished, due to low abundance and transcriptomic similarity to RGC and Imm-N populations. Notably, scRNA-Seq analysis (day 44 data set) revealed that 3D-default XFSC generated 61.2% D1-MSNs and 24% D2-MSNs among total cells, reaching 65.9% and 25.8%, respectively, among total neurons (Figure 3E), demonstrating efficient differentiation rate of MSNs in vitro.

We identified 3 distinct MSN subtypes, with each expressing canonical MSN markers (*FOXP2*, *PBX3*, and *TSHZ1* for D1-MSN1; *ZNF503*, *EBF1,* and *TAC1* for D1-MSN2; *SIX3*, *PENK*, and *MAFB* for D2-MSN) (Figure 3F and Supplemental Table 2), based on the top 100 differentially expressed genes (DEGs). Moreover, striosome (*KHDRBS3*, *TSHZ1*) and matrix markers express in D1-/D2-MSNs, suggesting early striosome or matrix specification (Supplemental Figure 4I). To investigate D1-/D2-MSN specification during in vitro differentiation, we performed DEG and Kyoto Encyclopedia of Genes and Genomes (KEGG) analyses to identify key genes or pathways potentially driving the divergence of D1-MSN and D2-MSN. The analysis identified many canonical D1- and D2-MSN markers as predominant DEGs (Figure 3G). Interestingly, based on the KEGG data analysis, circadian entrainment, morphine addiction, cholinergic synapses, serotonergic synapse, and dopaminergic synapse showed a stronger association with D1-MSN, whereas nicotine addiction was more closely linked to D2-MSN (Figure 3H).

Finally, we analyzed the similarity between in vitro–differentiated cells and those of the human fetal LGE (30). The results demonstrated a strong resemblance between D1-MSNs, D2-MSNs, and progenitors such as RGC and IPC obtained from in vitro cultures, and their corresponding cell types in vivo (Figure 3I). Moreover, 3D-default XFSC–derived day 20 progenitors resembled gestational week (GW) 12–13 LGE cells, and day 44 neurons matched in vivo counterparts from GW16 LGE (Supplemental Figure 4H).

Taken together, these results demonstrate that 3D-default XFSC efficiently generates LGE neural progenitors and MSN subtypes that closely resemble their fetal counterparts at single-cell resolution.

### Transplanted human LGE neural progenitors mature into D1- and D2-MSNs and project to endogenous targets in HD mice.

**Figure 4:**
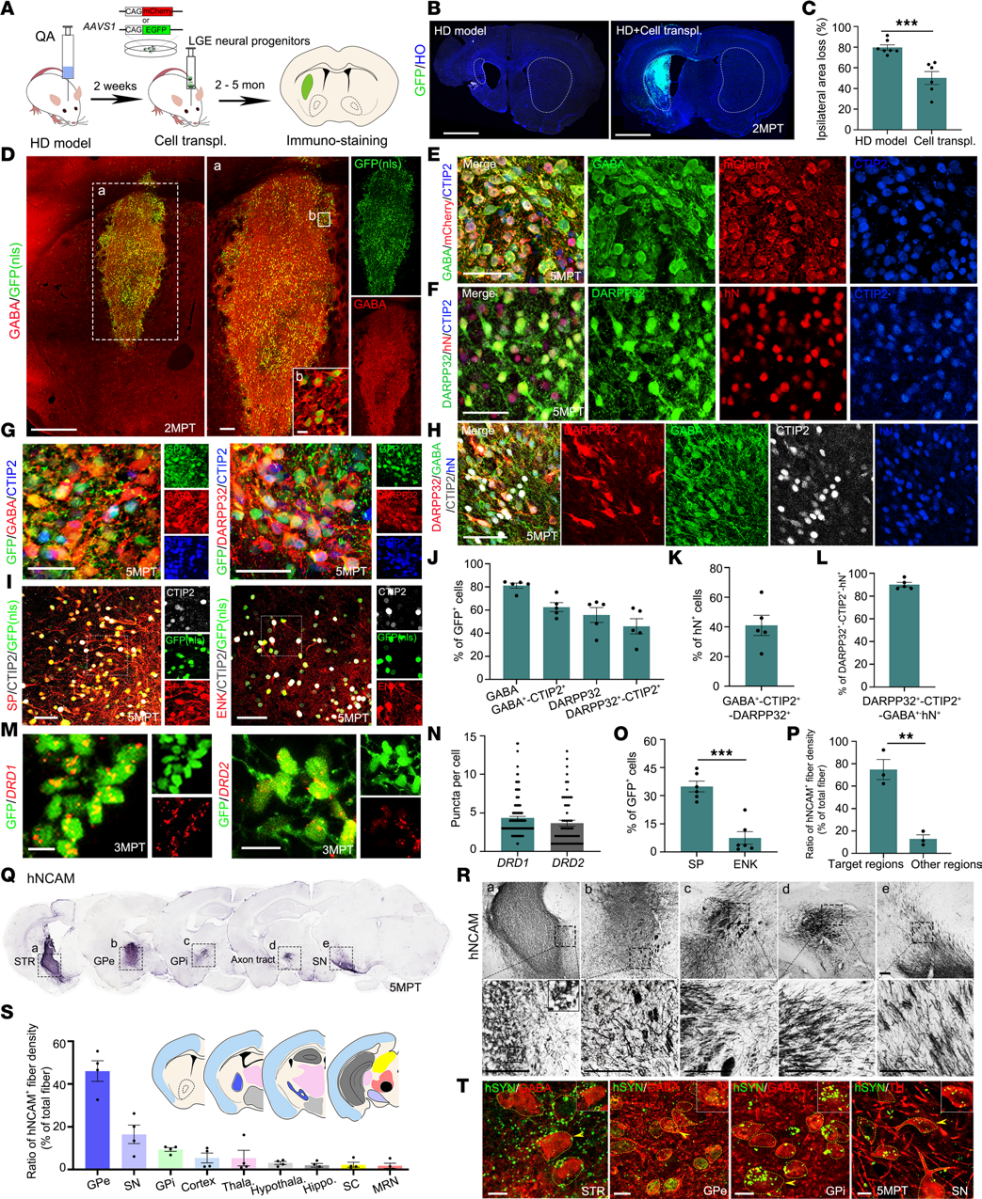
Neuron maturation and neural circuit reconstruction of grafted striatal neurons in HD mice. (**A**) The strategy of experimental design for cell transplantation (transpl.) in HD model mice. (**B** and **C**) Immunostaining (**B**) of GFP/Hoechst and quantification (**C**) of ipsilateral brain area loss in HD model mice with or without cell transplantation. Scale bar: 1 mm. (**D**) Representative images showing GFP (nuclear localization sequence [nls])/GABA double-labeling in grafts 2MPT. Scale bar: 500 μm. White dashed box is magnified in right panel (a). Scale bar: 100 μm. White box is magnified in lower panel (b). Scale bar: 10 μm. (**E** and **F**) Representative immunofluorescence images of GABA/mCherry/CTIP2 and DARPP32/hN/CTIP2 for grafts 5MPT. Scale bar: 50 μm. (**G**) Representative immunofluorescence images of GABA/GFP/CTIP2 and DARPP32/GFP/CTIP2 for grafts 5MPT. Scale bar: 25 μm. (**H**) Representative images of DARPP32/CTIP2/GABA/hN using tyramide signal amplification–based (TSA-based) immunofluorescence in grafts at 5 months after transplantation (5MPT). (**J**–**L**) Quantification of marker-positive cells among grafted cells. (**I** and **O**) Representative immunofluorescence images (**I**) and quantification (**O**) of SP^+^ and ENK^+^ cells in grafts 5MPT. Scale bar: 50 μm. (**M** and **N**) RNAscope labeling and quantification of *DRD1* and *DRD2* in the grafts 3MPT. (**P**) Quantification of hNCAM^+^ fibers in target areas (GPe, GPi, SN) and other brain regions. (**Q** and **R**) Immunohistochemical staining of hNCAM 5MPT. Boxed regions in (**Q**) show magnified views in (**R**). The black dashed box in **R** is further magnified in the lower panels. Scale bars: 100 μm. (**S**) Quantification of hNCAM^+^ fiber density in different brain regions. (**T**) Representative immunofluorescence images of presynaptic hSYN colocalized with host GABAergic neurons in the striatum, GPe, GPi, or with host TH^+^ dopaminergic neurons in the SN 5MPT. Hippo., hippocampus; Hypothala., hypothalamus; MRN, median raphe nucleus; SC, superior colliculus; Thala., thalamus. Scale bars: 10 μm. *n* = 3–7 mice. Data were analyzed with Student’s *t* test and presented as the mean ± SEM. **P < 0.01, ***P < 0.001.

To test if striatal progenitors generated by 3D-default XFSC survive and mature into MSNs and their subtypes, particularly in the HD-injured brain environment after transplantation, LGE neural progenitors derived from hESCs-H9-mCherry/-EGFP/EGFP–nuclear localization sequence) were dissociated and transplanted into QA-lesioned striatum of HD mice (Figure 4A and Supplemental Figure 5A). GFP immunostaining showed grafted striatal progenitors significantly reduced QA-induced striatal atrophy and ipsilateral ventricular enlargement compared with the contralateral side at 2 months after transplantation (2MPT) (Figure 4, B and C, and Supplemental Figure 5B). Only 3.82% ± 1.23% of grafted cells were KI67^+^ at 5MPT, indicating minimal proliferation of 3D-default XFSC–derived striatal progenitors in HD mice (Supplemental Figure 5, C and D). Immunostaining for GABA and GFP revealed that most GFP^+^ cells were GABAergic and evenly distributed within the graft, without graft overgrowth or aberrant migration at 2MPT (Figure 4D). Most grafted cells (81.12% ± 2.22%) were stained by GABA at 5MPT (Figure 4, G and J), indicating most 3D-default XFSC–derived LGE progenitors survived and differentiated into GABAergic neurons in HD mice. Notably, among grafted cells, 62.51% ± 3.85% co-expressed GABA and CTIP2, 55.71% ± 6.43% were DARPP32^+^, and 45.88% ± 6.57% showed DARPP32/CTIP2 co-localization, indicating MSN lineage (Figure 4, E–G, and J). More importantly, by tyramide signal amplification staining, we found 41.01% ± 6.79 % of grafted cells co-stained by DARPP32^+^-CTIP2^+^-GABA^+^, and greater than 90% of DARPP32^+^-CTIP2^+^ cells were GABAergic neurons (Figure 4, H, K, and L). Also, 34.89% ± 2.86% of grafted cells stained by SP, whereas 7.50% ± 3.36% were labeled with ENK at 5MPT (Figure 4, I and O). RNAscope staining revealed comparable densities of *DRD1*^+^ and *DRD2*^+^ puncta per cell in the grafts at 3MPT (Figure 4, M and N). Very few grafted cells were stained by markers for other cell types, including GABAergic interneurons (calretinin, parvalbumin, CHAT), astrocytes (GFAP), and oligodendrocyte (MBP) (Supplemental Figure 5, E and F). Together, these findings suggest that the transplanted LGE neural progenitors successfully developed into mature MSNs and their subtypes in HD mice.

The therapeutic effectiveness of the grafted cells depended on their capability for axonal projection and integration into the host brain. To investigate the neural circuit reconstruction by either D1- or D2-MSNs in HD brain, serial sections from 5MPT grafts were immunostained by human neural cell adhesion molecule (hNCAM). The results demonstrated that graft-derived human axons projected predominantly to their endogenous target areas: the GPe, GPi, and SNr, with obvious axon tracts between them (Figure 4Q, magnified views in Figure 4R). Quantification of hNCAM^+^ fiber density from representative coronal planes showed that 46.05% ± 4.83%, 9.39% ± 0.88%, and 16.49% ± 3.61% of hNCAM^+^ fibers were distributed in the GPe, GPi, and SN, respectively (Figure 4S), and very few were present in other brain regions such as cortex, thalamus, and hippocampus. We found that 74.81% ± 9.00% of hNCAM^+^ fibers from the graft projected to the targeted brain regions versus 13.99% ± 3.61% to other brain regions (Figure 4P).

Finally, to assess synaptic integration, human-specific SYN (hSYN) was immunostained with host neuronal markers. The results displayed obvious co-localization of hSYN with host GABAergic neuron in the striatum, GPe, GPi, and with host dopaminergic neurons (TH) in substantia nigra, demonstrating the establishment of synaptic connections between grafted cells and host neurons (Figure 4T). Collectively, these findings demonstrate that transplanted striatal MSNs extend projections to both GPi/substantia nigra and GPe, suggesting anatomic reconstruction of basal ganglia pathways in the HD mouse brain.

### Grafted human striatal progenitors developed into multiple subtypes of MSN at the single-cell transcriptional level in HD mice.

**Figure 5:**
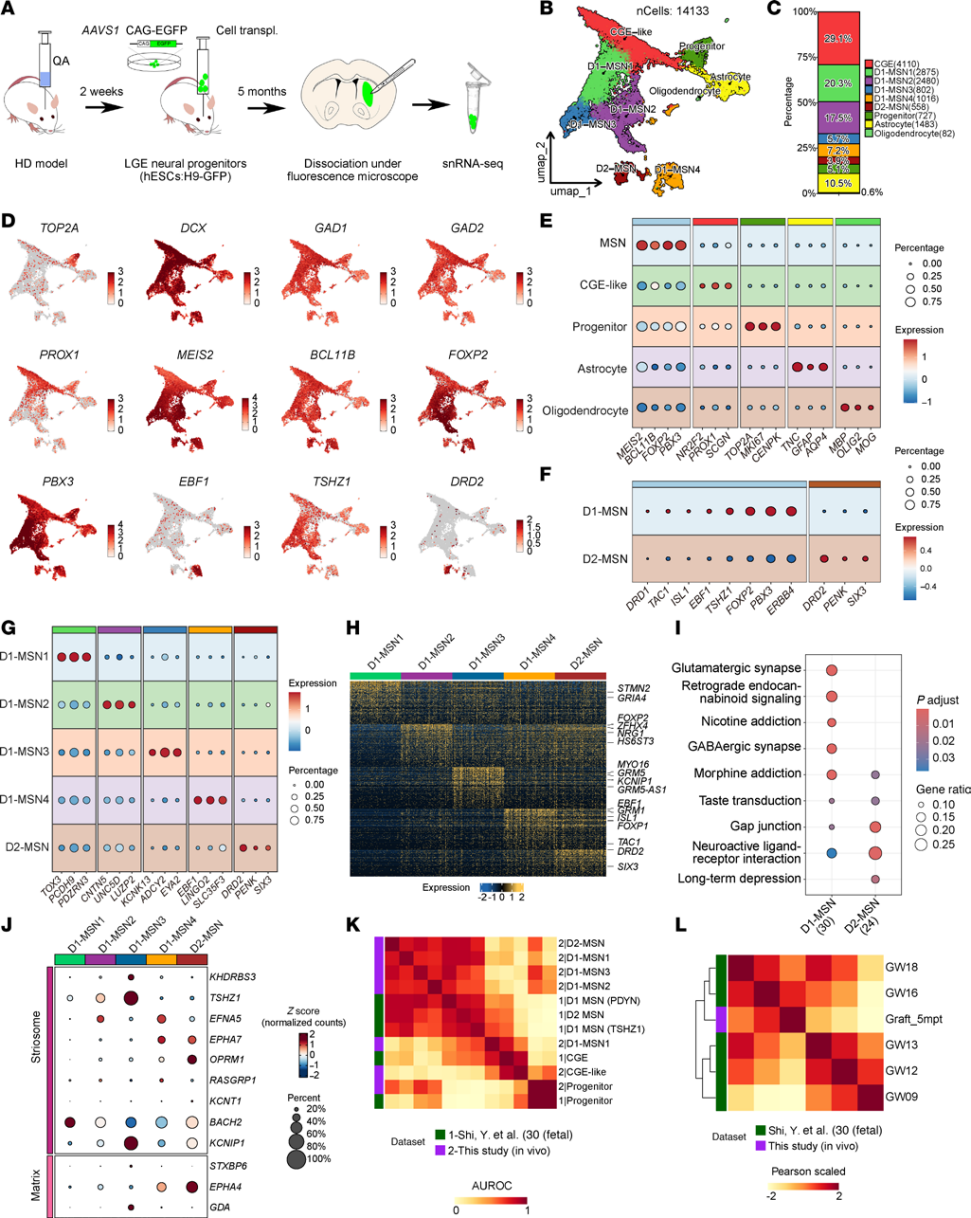
Characterization of human LGE progenitors and MSN subtypes after transplantation in HD mice using snRNA-Seq. (**A**) Schematic of the experimental design for cell transplantation in HD model mice and subsequent snRNA-Seq 5MPT. (**B**) UMAP visualization of all identified human cell clusters in the graft. (**C**) Bar plot showing the proportions of each cell type among total human cells. (**D**) UMAP plots displaying the expression of canonical marker genes for different cell types. (**E**) Dot plot showing the expression of marker genes for major cell types. (**F**) Dot plot showing the expression of canonical D1- and D2-MSN marker genes across the 2 main MSN subtypes. (**G**) Dot plot showing the expression of subtype-specific marker genes across all identified MSN subtypes. (**H**) Heatmap displaying the top 100 DEGs distinguishing MSN subtypes. (**I**) KEGG pathway enrichment analysis of DEGs in D1-MSNs versus D2-MSNs. (**J**) Dot plot showing the expression of marker genes associated with striosome and matrix among MSN subtypes. (**K**) Heatmap depicting pairwise transcriptional correlations of 3D XFSC–derived progenitors and MSNs in the grafts with those in the developing human brain data set (30). (**L**) Heatmap depicting pairwise transcriptional correlations of graft-derived cells and developmental stages of human brain data set (30).

To investigate the characteristics of grafted cells differentiated by 3D-default XFSC in HD mice, we conducted single-nucleus RNA-Seq (snRNA-Seq) at 5MPT (Figure 5A). After quality control, a total of 14,133 human cells were collected. Unsupervised method was used to cluster cells, and we detected 9 cell clusters that annotated by canonical markers (Figure 5, B and E, and Supplemental Table 3). UMAP visualization and the bar plot revealed that, except for small population of astrocytes and oligodendrocytes (Figure 5C), the majority of cells expressed *DCX*, *GAD1*, and *GAD2*, and key genes for MSN development (*MEIS2* and *BCL11B*) (Figure 5D), indicating identity of striatal GABAergic neurons. These neurons were further classified into MSN subtypes based on subtype-specific transcription factors *TAC1*, *ISL1*, *EBF1*, *FOXP2*, and *PBX3* for D1-MSN, and *DRD2* and *SIX3* for D2-MSN (Figure 5, D–F).

Based on transcriptomic heterogeneity, D1-MSN can be further subdivided into 4 distinct subclusters (D1-MSN1–4), each exhibiting unique marker gene expression profiles and representing distinct cellular proportions (20.3%, 17.5%, 5.7%, and 7.2% for D1-MSN clusters 1–4, respectively) (Figure 5, C and G). Some of these subtype-specific genes were also enriched as DEGs between MSN subtypes (Figure 5H and Supplemental Table 4). Notably, we identified a cell population in 5-month grafts that highly expressed CGE signature genes (*NR2F2*, *PROX1*, *SCGN*) but lacked expression of GABAergic interneuron markers (Figure 5, B–E, and Supplemental Figure 6E), suggesting these might represent an intermediate cell state (named CGE-like cells). Moreover, minimal expression was detected for MGE-lineage markers (*NKX2.1*, *LHX6*) and glutamatergic neuron–associated genes (*NEUROD2*, *SLC17A6*, *SLC17A7*, *TBR1*) (Supplemental Figure 6E).

These results demonstrate that striatal neural progenitors differentiated via 3D-default XFSC can mature into transcriptionally distinct MSN subtypes at 5MPT in HD mice. Notably, although scRNA-Seq of 3-month grafts revealed less maturity compared with 5-month grafts, they demonstrated strong transcriptional concordance and clustering alignment with their later-stage counterparts (Supplemental Figure 6, A–D), indicating methodological reproducibility.

Interestingly, KEGG analysis of DEGs enriched distinct pathways between MSN subtypes. D1-MSN strongly associated with glutamatergic/GABAergic synapse formation and reward-processing pathways (namely, retrograde endocannabinoid signaling, nicotine addiction, and morphine addiction), whereas D2-MSNs were preferentially associated with gap junction signaling, neuroactive ligand-receptor interactions, and long-term depression pathways (Figure 5I). Marker genes for striosome (*TSHZ1, BACH2, KCNIP1*) and matrix (*EPHA4*) were detected in D1- or D2-MSNs, indicating the participation of both MSN subtypes in the formation of matrix and striosome (Figure 5J). More importantly, similarity scores between data from the 5-month graft and human fetal brain indicated a high transcriptional similarity between the grafted human cells and the in vivo counterparts, particularly D1-MSN and D2-MSN cell clusters (Figure 5K). The 5-month graft also exhibited high similarity with human fetal striatal tissue from GW16–18 (Figure 5L), indicating cell-intrinsic maturation programs of human neurons in mice.

Together, these findings illustrate that neural progenitors derived from 3D-default XFSC efficiently differentiated into various MSN subtypes that closely resembling the human fetal striatum at the single-cell resolution after transplantation in HD mice.

### Transplanted human MSNs have a characteristic response to DA in HD mouse brain.

**Figure 6:**
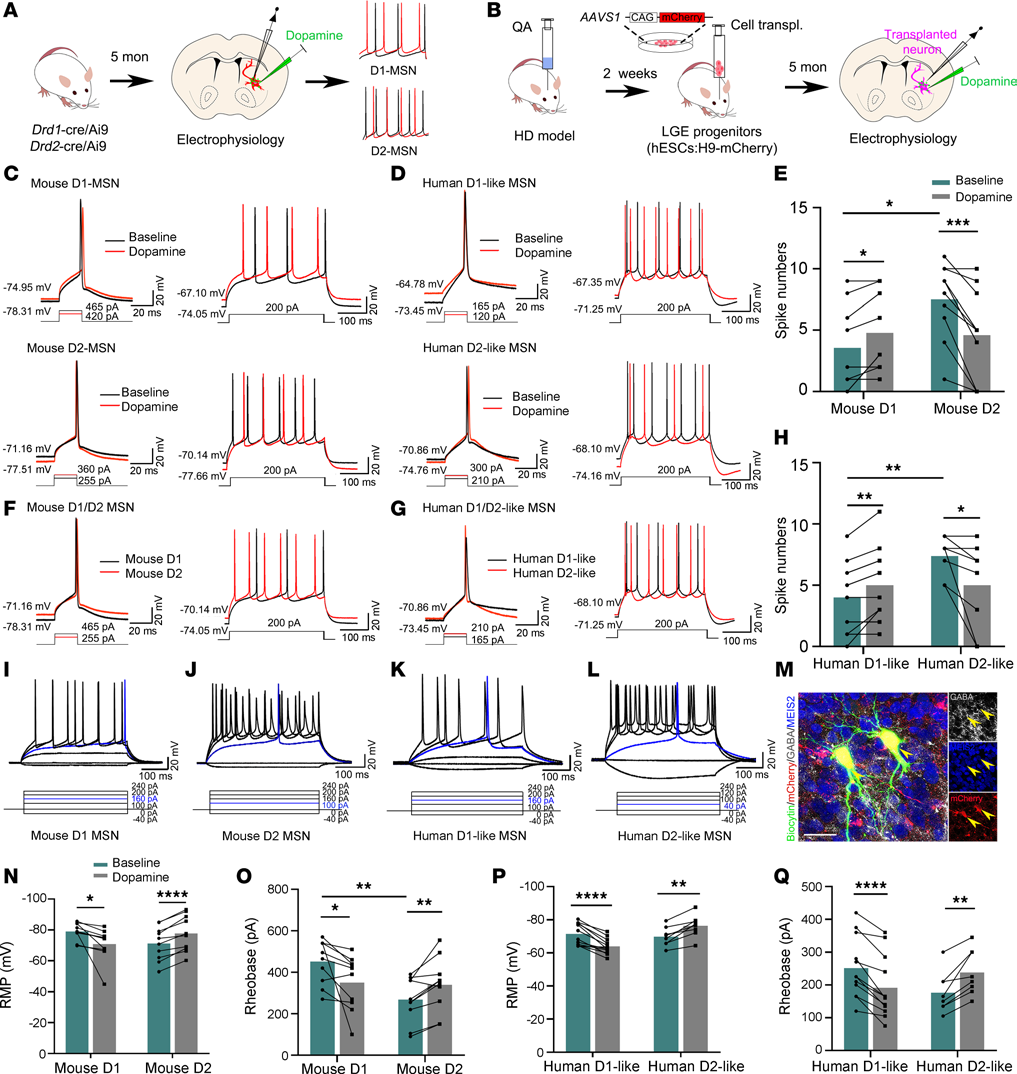
Neuromodulation of striatal MSN by DA in transgenic mice and human graft in HD mice. (**A** and **B**) The strategy for electrophysiological recording in transgenic mice (**A**) and in HD mice with human graft (**B**). (**C**) Current-induced single and multiple AP traces from D1-MSNs and D2-MSNs in transgenic mice under baseline conditions and after DA application. (**D**) Current-induced single and multiple AP traces in human D1-like MSNs and D2-like MSNs from grafted neurons 5MPT, under baseline conditions and after DA application. (**E** and **H**) Quantification of DA-induced changes in spike numbers evoked by depolarizing pulses: for transgenic mice, 240 pA for D1-MSNs (*n* = 9 cells from 5 mice), 200 pA for D2-MSNs (*n* = 10 cells from 4 mice); for grafted neurons, 100 pA for D1-like MSN (*n* = 12 cells from 6 mice), 120 pA for D2-like MSN (*n* = 8 cells from 6 mice). (**F** and **G**) Traces of current-induced single and multiple APs for both D1-MSNs and D2-MSNs from transgenic mice (**F**), and human D1-like MSNs and human D2-like MSNs from grafts 5MPT (**G**). (**I**–**L**) Traces from selected step current injections showing near-threshold stimuli elicited delayed spiking in D1-/D2-MSNs in mice, and D1-like/D2-like MSNs in 5-month human grafts. (**M**) Electrophysiological recorded neurons were stained by biocytin, mCherry, MEIS2, and GABA. Scale bar: 50 μm. (**N**–**Q**) Statistical analyses of the RMP (**N**) and rheobase (**O**) in D1-/D2-MSNs from transgenic mice, or human D1-like/D2-like MSNs in 5-month human grafts (**P** and **Q**), before and after DA application (*n* = 10 cells from 5 mice for mouse D1-MSN; *n* = 10 cells from 4 mice for mouse D2-MSN; *n* = 12 cells from 6 mice for human D1-like MSN; *n* = 8 cells from 6 mice for human D2-like MSN). Paired Student’s *t* tests compared before and after DA treatments; unpaired Student’s *t* tests analyzed between-group differences. Data are presented as the mean ± SEM. *P < 0.05, **P < 0.01, ***P < 0.001, ****P < 0.0001. transpl., transplantation.

Previous studies demonstrated distinct electrophysiological divergence between D1- and D2-MSNs in response to DA (31, 32). To examine the subtype-specific functional responses of grafted human MSNs to DA in HD mice, we detected the electrophysiological properties of native mouse D1- and D2-MSNs, using *Drd1*- and *Ddr2*-cre/Ai9 transgenic mice first (Figure 6A). We injected depolarizing current steps to evoke APs and analyzed firing patterns, both at baseline and during DA application (Figure 6, C and F). By analyzing single APs, we found that the RMP of D1-MSNs (–78.93 ± 1.73 mV) was more hyperpolarized relative to that of D2-MSNs (–71.25 ± 3.46 mV). DA stimulation resulted in a significant increase of the RMP in D1-MSNs, whereas a decrease in the RMP was observed in D2-MSNs (Figure 6N). Furthermore, the rheobase, defined as the minimum current required to elicit an AP, was considerably greater in D1-MSNs than in D2-MSNs (D1: 451.50 ± 33.43 pA; D2: 268.00 ± 34.02 pA), and DA application markedly reduced the rheobase in D1-MSNs (350.00 ± 41.41 pA) but substantially increased it in D2-MSNs (339.5 ± 41.13 pA) (Figure 6O). Moreover, the neuronal excitability of D1- and D2-MSNs was also revealed by AP spike numbers evoked by graded depolarizing current injections (0–320 pA; step: 20 pA; duration: 400 ms). We found DA administration oppositely regulated MSN excitability in response to identical current steps: D1-MSNs showed increased firing (from 3.56 ± 1.17 to 4.78 ± 1.08 spikes at 240 pA), whereas D2-MSNs exhibited decreased firing (from 7.50 ± 0.98 to 4.60 ± 1.18 spikes at 200 pA) (Figure 6, C, E, and F). These findings suggest DA enhances excitability in D1-MSNs while reducing it in D2-MSNs in transgenic reporter mice.

Based on the distinctive responses to DA observed in endogenous D1- and D2-MSNs, we next investigated whether transplanted neurons can similarly exhibit subtype-specific responses to DA modulation. To trace grafted cells, we transplanted LGE neural progenitors derived from H9-mCherry into the striatum of HD mice and recorded their functional properties by electrophysiology at 5MPT (Figure 6B). Except for 4 cells that did not respond, we recorded a total of 20 transplanted human neurons. Remarkably, we observed D1-like MSNs and D2-like MSNs with similar electrophysiological characteristics in AP and spike firing patterns compared with mice (Figure 6, D and G). In particular, compared with D2-like MSNs, which had an average RMP of –69.76 ± 2.04 mV, the RMP of D1-like MSNs was lower, measuring –71.46 ± 1.89 mV. When DA was applied, significant changes were observed: the RMP of D1-like MSNs increased to –63.92 ± 1.35 mV, whereas that of D2-like MSNs decreased to –76.36 ± 2.35 mV (Figure 6P). Furthermore, after the application of DA, the rheobase was dramatically reduced from 251.30 ± 26.77 to 187.5 ± 28.84 pA in D1-like MSNs, whereas in D2-like MSNs, the rheobase exhibited a substantial increase from 176.30 ± 21.91 to 238.1 ± 24.29 pA (Figure 6Q). Graded depolarizing current injections (0–320 pA; 20 pA steps; 400 ms duration) revealed that DA administration exerted subtype-specific effects on grafted neurons: D1-like MSNs had increased AP numbers (from 4.00 ± 0.81 to 5.00 ± 0.86 spikes, at 100 pA), whereas D2-like MSNs had decreased firing (from 7.38 ± 0.57 to 5.00 ± 1.25 spikes, at 120 pA) (Figure 6, D, G, and H). Among 20 recorded cells, 60% (*n* = 12) had D1-like characteristics (increased firing), and 40% (*n* = 8) exhibited D2-like properties (decreased firing) after DA application. Notably, in whole-cell recordings of voltage responses to depolarizing current steps, both native mouse MSNs and human MSN-like neurons exhibited obvious late-spiking pattern responses to near-threshold stimuli (blue line in Figure 6, I–L)—a typical MSN firing pattern due to type-A potassium currents (33, 34). The current step sufficient to elicit a single spike in a D2-MSN was not strong enough to trigger spike in a D1-MSN, suggesting high excitability of D2-MSNs compared with D1-MSNs (Figure 6, I–L). The recorded human neurons were stained positive for biocytin, GABA, MEIS2, and mCherry (Figure 6M).

In addition, comparable parameters, including peak amplitude and AP threshold, were significantly different between D1- and D2- MSNs in either mouse or human cells (Supplemental Figure 7, A and B). DA treatment revealed no significant differences in input resistance between D1- and D2-MSNs within species; however, human MSNs exhibited higher input resistance than mouse MSNs (Supplemental Figure 7C), possibly due to different cell size or ion channels of human neurons versus mouse. We also found that membrane capacitance did not significantly differ between mouse and human (Supplemental Figure 7D). However, the half-width of the AP in human D1-like MSNs was significantly longer than that of mouse D1-MSNs (Supplemental Figure 7E).

Meanwhile, the kinetic curves of transplanted neurons, particularly human D1-like MSNs had a significantly reduced maximum rise and decay slope, along with longer rise and decay times, compared with their mouse counterparts (Supplemental Figure 7, F–J). These electrophysiological differences may reflect variations in ion channel properties or maturation states between mouse and human.

To test the passive electrophysiological properties of MSNs among different subtypes, a series of increasingly negative current pulses were injected into individual neurons (Supplemental Figure 7, K and L). Interestingly, consistent with previously reported findings (24), we observed human MSN-like neurons exhibit sag and rebound like responses (Supplemental Figure 7L) to hyperpolarizing current steps, suggesting potential roles of h-conductance in human developing MSNs.

Overall, these findings demonstrate that transplanted LGE neural progenitors successfully differentiate into functional neurons exhibiting electrophysiological properties akin to endogenous D1- and D2-MSNs.

### Grafted neurons integrated into host neural circuit and restored motor function of HD mice.

**Figure 7:**
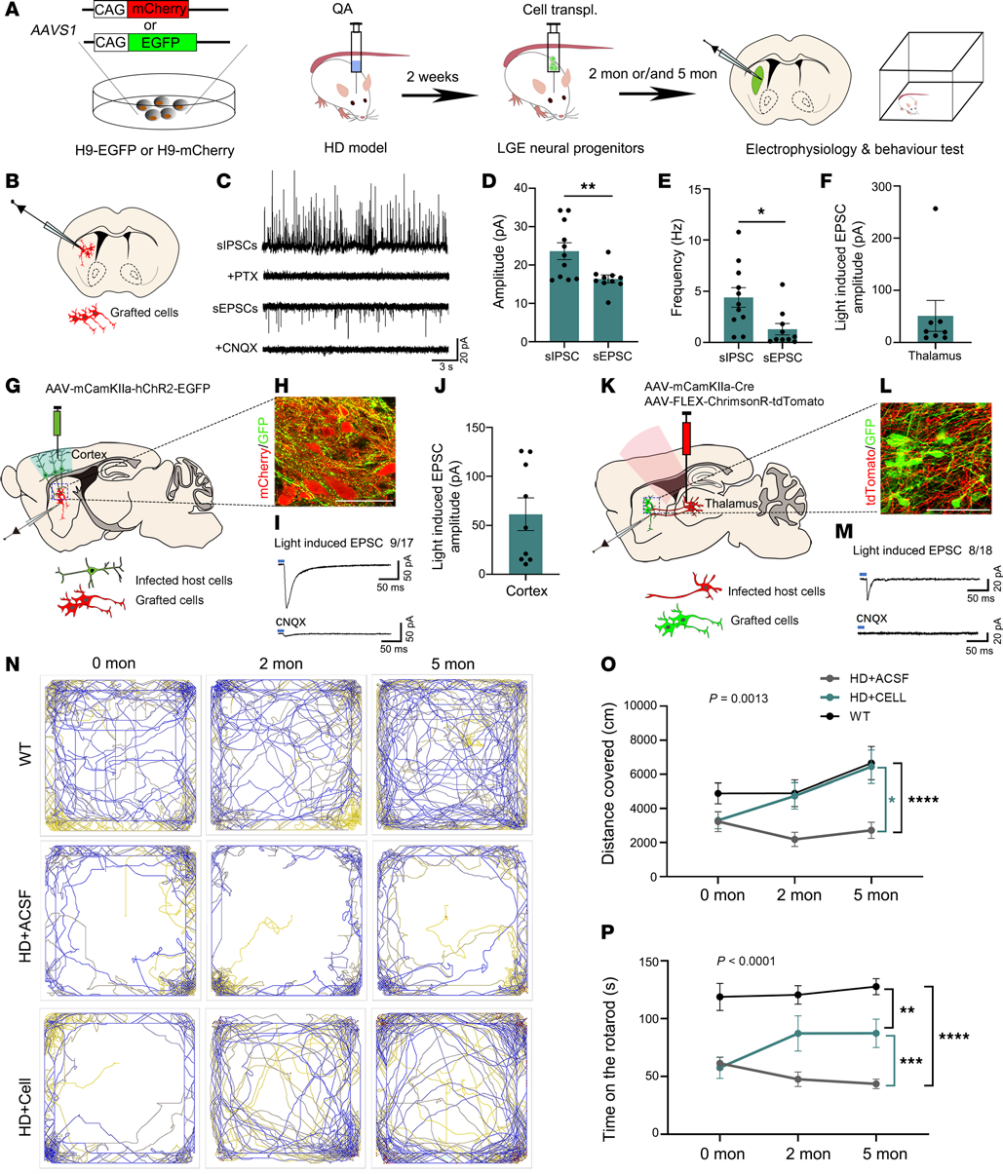
Grafted striatal neuron integrated into host neural circuit and alleviated motor defects of HD mice. (**A**) Experimental design for cell transplantation (transpl.) and behavioral tests. (**B**–**E**) Schematic of electrophysiological recordings and representative sIPSC/sEPSC traces of grafted neurons 5MPT, with quantified amplitude and frequency. Data were analyzed by Student’s *t* test. (**G**) The strategy for whole-cell patch-clamp recording of synaptic regulation from host cortex. (**H**) Representative image showing host cortical GFP^+^ fibers around mCherry^+^ grafted neurons. (**I**) Typical traces showing light-evoked EPSCs in grafted neurons 5MPT (top) and the subsequent blockade by CNQX (bottom). (**J**) Quantified amplitudes of optogenetically induced EPSCs (*n* = 9 cells from 3 mice). (**K**) The strategy for whole-cell patch-clamp recording of synaptic regulation from host thalamus. (**L**) Representative image of host thalamic tdTomato^+^ fibers around GFP^+^ grafted neurons. (**M** and **F**) Light-evoked EPSCs in grafted neurons (blocked by CNQX) with amplitude quantification (*n* = 8 cells from 3 mice). (**N** and **O**) Representative open-field movement traces and total distance covered by WT, ACSF-injected, and striatal neuron-grafted HD model animals at 0MPT, 2MPT, and 5MPT. (**P**) The rotarod test showing time of latency to fall in different groups at 0MPT, 2MPT, and 5MPT. *n* = 8–10 mice for HD+ASCF, 8–13 mice for HD+Cell, and 6–13 mice for WT. Two-way ANOVA was used for comparison between different groups. Data are presented as the mean ± SEM. *P < 0.05, **P < 0.01, ***P < 0.001, ****P < 0.0001.

To investigate whether the grafted neurons could integrate into the host neural circuit functionally, LGE neural progenitors derived from hESC-H9-EGFP/-mCherry were transplanted into the ipsilateral striatum of the HD mice (Figure 7A). Patch-clamp recordings of grafted neurons (5MPT) exhibited sIPSCs and excitatory post-synaptic currents (sEPSCs), which were blocked by the GABA_A_ receptor antagonist PTX and AMPA receptor antagonist CNQX, respectively, indicating synaptic integration (Figure 7, B and C). Higher sIPSC amplitudes and frequencies were observed compared with sEPSCs (Figures 7, C–E), indicating more inhibitory than excitatory synaptic inputs for grafted neurons. To further characterize host-to-graft regulation anatomically and functionally, HD mice with human striatal progenitor transplantation were injected with AAV-mCamKIIa-hChR2-EGFP into motor cortex, or AAV-mCamKIIa-Cre/AAV-FLEX-ChrimsonR-tdTomato into the thalamus at 5MPT (Figures 7, G and K). Numerous GFP^+^ fibers originating from cortical projections were observed surrounding the grafted neurons (mCherry^+^) in the striatum (Figure 7H), indicating establishment of anatomic connectivity between host cortex and grafted neurons. Blue-light stimulation of these cortical GFP^+^ fibers elicited obvious EPSCs in grafted neurons, an effect that was blocked by CNQX (Figure 7, I and J). Similarly, optogenetic stimulation of host thalamic neuron fibers evoked robust EPSCs in the grafted cells, around which abundant thalamic axon terminals were observed. (Figure 7, F and K–M). These findings demonstrate functional excitatory synaptic inputs from host cortical and thalamic neurons onto the transplanted cells.

Finally, to determine whether the grafted neurons could rescue behavioral deficits in HD model mice, animals were subjected to assessments for motor behavior tests, including open field and rotarod tests before transplantation(0 months), and 2 and 5 months after transplantation (Figure 7, N–P). Those injected with artificial cerebrospinal fluid (ACSF) and unlesioned WT animals were used as controls. In the open field test, the trajectory map and quantitative analysis revealed that transplanted HD animals traveled a significantly increased total distance compared with ACSF controls, with performance even approaching that of WT animals at both 2 and 5 months after transplantation (Figure 7, N and O). The motor coordination and balance assessment through the rotarod test demonstrated increased latency to fall compared with ACSF-treated controls. Nevertheless, this improvement remained below WT control levels at both 2MPT and 5MPT (Figure 7P), indicating partial recovery of motor coordination by grafted neurons at this time point (Figure 7P).

Together, these data indicate that grafted striatal neurons generated by 3D-default XFSC integrated into host neural circuits and restored motor deficits in HD model mice in the long term.

## Discussion

Dysfunction of striatal MSNs is involved in motor, cognitive, and psychiatric symptoms (33). Generating MSNs is a powerful tool for dissecting the mechanisms of MSN-related disorders and developing therapeutic therapies, but few studies have obtained authentic MSNs, particularly D1 and D2 subtypes, regarding histological, transcriptional, and functional properties in vitro and in vivo (14). In this research, the 3D-default XFSC method produced LGE progenitors and MSN subtypes that faithfully mimic crucial transcriptional features of their native counterparts. Notably, these cells differentiated into multiple MSN subtypes, integrated into the host neural circuit, and successfully restored functionality in an HD model mouse after transplantation.

Currently, a range of protocols targeting different signaling pathways, including SHH, WNT, or TGF-β, have been applied to promote the production of LGE-derived MSNs. However, inconsistent differentiation efficiency and therapeutic efficacy of these hPSC-derived MSNs were observed across studies, highlighting the ongoing challenges in achieving consistent, high-quality MSN generation for stable therapeutic effects (14). Based on developmental principles, dorsal-ventral patterning is regulated by opposing signaling gradients. SHH mediates ventral specification, whereas WNT/BMP signaling orchestrates dorsal patterning (35). Although some research advocates that a specific level of SHH signaling is advantageous for the generation of MSNs (14), other studies have noted that the addition of exogenous SHH is dispensable for the generation of LGE-like progenitors (10, 13, 36, 37). In this study, we observed sequential, time-dependent expression of SHH signaling components (SHH, GLI1) and key transcription factors for striatum development (MEIS2, GSH2) during neural differentiation (Figure 1 and Supplemental Figure 1), demonstrating that exogenous SHH supplementation may be largely dispensable for striatal cell differentiation in 3D culture systems, because endogenous ventralizing signals appear sufficient to promote LGE cell fate. Intriguingly, although Western blot analysis revealed significantly lower SHH protein levels in neurospheres (day 7) generated by 2D-default EB compared with 3D-default culture, SHH expression was still detectable by immunofluorescence (Figure 1, J and O). However, the SHH pathway effector GLI1 was not observed in 2D-default EB-derived neural progenitors. Previous studies have demonstrated that 2D-default EB neural differentiation activates endogenous WNT signaling, which upregulates GLI3R and consequently inhibits SHH pathway activity (27). In this study, we observed low WNT levels in 3D-default–derived neurospheres (Figure 1I), likely preventing GLI3R-mediated suppression of SHH signaling. Thus, we propose that 3D-default XFSC drives LGE fate commitment by concomitantly enhancing SHH signaling and repressing the WNT pathway.

The requirement for ventralizing factors in LGE neuron differentiation could be culture-system dependent, given that folding neural tube–like structures emerged in feeder-free hESC-derived spheres under 3D-default conditions (but not in 2D cultures) during early neural differentiation (Supplemental Figure 1, H–J). This morphological difference may promote activation of different intrinsic signaling pathways that fundamentally reshape neural induction programs. This paradigm is gaining experimental support, particularly in striatal differentiation, where 3D organoid protocols achieve robust ventralization with minimal exogenous SHH (38, 39), in contrast to 2D systems that typically require high morphogen exposure (9, 22). However, the mechanistic basis for reduced WNT signaling in 3D-default XFSC versus 2D-default EB cultures remains to be elucidated.

The selective loss of striatal MSNs in HD (40) make them prime candidates for cell replacement therapy (12, 14). Traditional quality control measures relying on limited markers like CTIP2 and DARPP32 fail to fully capture MSN authenticity, with studies consistently reporting decreased DARPP32 expression after transplantation compared with in vitro conditions (41). We analyzed 3D-default XFSC–derived MSNs using advanced histology and single-cell transcriptomics to assess developmental fidelity. Immunostaining confirmed consistent expression of pan-MSN markers CTIP2, MEIS2, GABA with DARPP32, and subtype-specific markers (FOXP2 and SP for D1; SIX3 and ENK for D2) in vitro and in vivo. Single-cell sequencing not only validated these findings transcriptionally but also revealed striking similarity to human fetal MSNs (30), indicating the well-defined and authentic identity of human MSNs derived by 3D-default XFSC. Notably, consistent with prior scRNA-Seq data from GW7–GW18 fetal LGE (30, 42), minimal *DRD1* and *DRD2* expression was observed by snRNA-Seq in 5-month grafts. We propose reduced sensitivity of snRNA-Seq for low-expression genes (43), given that *DRD1/DRD2* was detected by a more sensitive technique (RNAscope) in this study. In addition, whereas most markers exhibited a consistent proportion between in vitro and in vivo conditions after transplantation, we observed an unexpected reversal in MSN subtype proportions: there were more D2-MSNs than D1-MSNs in vitro, and the opposite was observed in vivo, as revealed by SP and ENK staining (Figure 2, T and V, and Figure 4, I and O). This could be associated with the susceptibility of D2-MSNs compared with D1-MSNs in stress or disease environments (28, 44). Interestingly, scRNA-Seq analysis demonstrated that genes critical for specific synapse formation were differentially enriched across MSN subtypes, both in vitro and in vivo (Figure 3H and Figure 5I), indicating that developing MSNs are transcriptionally preprogrammed with subtype-specific connectivity biases prior to synaptogenesis. These findings are consistent with a previously discovered phenomenon in mice that distinct MSNs display specific circuit preference (45).

In this study, we observed that transplanted striatal neurons extended projections to endogenous striatal target areas (GPe, GPi, and SN), with apparent regional preference compared with other brain regions, indicating their capacity to navigate toward appropriate targets. However, it remains unclear whether these projections have subtype specificity, that is, whether D1-MSNs primarily project to the GPi and SN, whereas D2-MSNs mainly target GPe. In addition, because the cells were transplanted into the striatum, adjacent to target areas, particularly GPe, the observed innervation within the GPe might partially result from anatomic proximity rather than selective target recognition. Future studies should combine D1/D2-MSN–specific reporters with retrograde tracing to verify subtype-specific projection targeting. Moreover, the grafted cells received both excitatory and inhibitory synaptic inputs and ameliorated motor deficits of HD animals (Figure 7). Nevertheless, more inhibitory, but less excitatory, inputs were observed in grafted neurons (Figure 7D). We propose that host-to-graft synaptic connectivity remains under development, resulting in few mature excitatory inputs to the grafted neurons at 5MPT, due to intrinsic neotenic properties of human neurons (46), Meanwhile, inhibitory connections between transplanted striatal GABAergic neurons within the graft could also result in more inhibitory inputs for grafted neurons. Notably, the frequencies of excitatory (~2 Hz) and inhibitory (~5 Hz) postsynaptic currents in grafted striatal neurons align with those observed in other study 5-6 months post-transplantation (12), suggesting that grafted neurons may require extended maturation periods to establish robust excitatory innervation.

Dopaminergic innervation of MSNs generally regulates membrane excitability through DA receptors. Specifically, DA activation of D1 receptor increases D1-MSN excitability, whereas D2 receptor stimulation suppresses D2-MSN activity (47). In this study, the classification of D1-like and D2-like MSNs was primarily based on electrophysiological responses to DA, including RMP, rheobase, and neuronal excitability, which were inferred through comparisons with established mouse cell properties. Although these electrophysiological properties are widely accepted indicators of D1- and D2-MSN phenotypes across species (48, 49), more definitive classification of these grafted human neuronal populations as bona fide D1- or D2-MSNs would require either transcriptomic profiling of marker genes expression through Patch-Seq or pharmacological validation via subtype-specific receptor blockade. Our results also indicate that the electrophysiological properties of these human neurons, although still transcriptionally immature, do exhibit remarkable similarities to mature mouse neurons in some aspects, particularly in their response to DA stimulation. This finding was also observed in previous studies that DA and functional DA receptors are present during early neural development in the brain prior to the onset of synaptogenesis (29, 50). Nevertheless, the transplanted human neurons do not fully replicate the electrophysiological profile of mature mouse MSNs in all aspects. Specifically, we observed human D1-like/D2-like MSN present significant higher input resistance, longer AP half-width and slower kinetics of membrane potential dynamics, particularly in human D1-like MSN, compared to their mouse counterparts (Supplemental Figure 7, C and E–J). These discrepancies likely reflect the complex interplay of multiple ion channels involved in AP generation and the protracted maturation process of human neurons. Furthermore, considering MSN differentiation efficiency in vivo and the neoteny of human neurons compared to that of rodents (46), comprehensive functional analysis of grafted human MSNs will require both longitudinal electrophysiological recordings at multiple post-transplantation time points and the use of DARPP32-/DRD1-/DRD2-GFP reporter lines for reliable MSN subtype identification.

Numerous studies have successfully grafted MSN-like neurons, astrocytes, or immortalized neural stem cell lines derived from hPSCs, demonstrating induced neuroprotection and functional recovery in animal models of HD (41, 51–53). Of note, the adult striatum comprises heterogeneous glia cells and neuronal cells, of which 90%–95% are MSNs (the proportion is 80%–85% in primates). In rodents, 5%–10% are interneurons; this proportion is up to 20% in primates (54). Fetal striatal grafts, comprising a heterogeneous population of cells including MSNs, interneurons, and glia, have more robust functional recovery and integration (55), indicating the presence of diverse cell types, including glia, is crucial for the optimal survival and function of transplanted human neurons. In this study, snRNA-Seq data identified 11.1% glia and 29.1% CGE-like cells in grafts (Figure 5, B and C), potentially supporting graft survival and integration in HD mice. However, the relatively lower glia-to-neuron ratio in mouse versus human striatum may limit grafted human neuron survival and differentiation in this model (56, 57). Thus, the therapeutic efficacy of cell transplantation into different species of host brains may vary, increasing the complexity of HD cell therapy and highlighting the importance of multispecies preclinical testing. Additionally, because QA injection leads to an excitotoxic lesion with extensive MSN loss, differing from the gradual neuronal loss observed in patients with HD (58, 59), it is important to conduct further evaluations of the functionality of grafted human striatal neurons, using genetic HD models.

In conclusion, our research offers an efficient strategy for producing authentic and scalable MSNs and promoting standardization and quality control during manufacturing. Importantly, the grafted MSNs projected to native targets formed synaptic connections with host cells, responded to DA modulation appropriately, and successfully restored motor defects in an HD mouse model, indicating the promising potential for using these cells in future applications.

## Methods

### Sex as a biological variable.

Sex was not considered a biological variable.

### Experimental animals.

SCID beige mice (male and female) were purchased from Vital River Laboratories. All animals were group housed under a 12-hour light/dark cycle with free access to food and water.

### Statistics.

Bright-field images of mouse brain slices were captured using an Olympus VS200 microscope at an original magnification of ×20. Fluorescence images were captured on a Leica SP8, Nikon NSPARC, Nikon AX microscope, or Nikon TIE microscope (×20 or ×60 magnification). All experiments for cell culture were independently repeated 3 times. Positive cells were manually quantified using ImageJ (NIH). GraphPad Prism (GraphPad Software, La Jolla, CA) was used for statistical analysis. Each data point in the bar graphs represents a field of view for cell counts, an individual electrophysiologically recorded cell, or behavioral data from a single animal. The results were analyzed by 2-tailed Student’s *t* test, 1-way ANOVA, or 2-way ANOVA. The *P* values for the ANOVA main effects reported in the figures. A *P* value of less than 0.05 was considered significant.

### Study approval.

All animal studies were conducted according to a protocol approved by the Experimental Ethics Committee at the School of Basic Medical Science, Fudan University (approval no. 20220228-159, February 28, 2022; project title: Stem cell therapy for brain disorder). These experiments were performed in accordance with the NIH Guidelines for the Care and Use of Laboratory Animals.

### Data and code availability.

The raw sequence data reported in this article have been deposited in the Genome Sequence Archive (GSA) in the National Genomics Data Center, China National Center for Bioinformation/Beijing Institute of Genomics, Chinese Academy of Sciences (GSA-Human HRA011498) and are publicly accessible at https://ngdc.cncb.ac.cn/gsa-human Further information and code can be requested from the corresponding authors. All data associated with this study are present in the article, the supplemental figures, and the Supporting Data Values file.

Detailed methods are included in the Supplemental data.

## Author contributions

MX conceived and supervised the project. MX and YC designed the research studies and conducted experiments. YM and YX performed cell differentiation and transplantation, histology experiments, behavioral tests, and the statistical analysis. YM, XZ, and L Xie performed scRNA-Seq experiments and bioinformatics analyses. BF and YZ performed electrophysiological recordings. YL, XP, QC, L Xiao, MW, and WZ helped with animal experiments and data analysis. YM and MX wrote the manuscript. YM, YC, and MX edited the manuscript. The order of the authors was assigned on the basis of their contributions to the study.

## Supplementary Material

Supplemental data

Unedited blot and gel images

Supplemental table 1

Supplemental table 2

Supplemental table 3

Supplemental table 4

Supplemental table 5

Supporting data values

## Version 1 10/15/2025

Electronic publication
