# Supplementary material for: 3D cultured human medium spiny neurons functionally integrate and rescue motor deficits in Huntington’s disease mice
Source: J Clin Invest. 2025 Oct 15;135(20):e187941. doi: 10.1172/JCI187941 (PMC12520685; doi:10.1172/JCI187941)
Supplement: Supplemental data [file jci-135-187941-s148.pdf]

SUPPLEMENTAL MATERIALS

3D-cultured human medium spiny neurons functionally integrate and rescue motor deficits in Huntington's disease mice

Yuting Mei[1, 2, #], Yuan Xu[1, #], Xinyue Zhang[3, 4, #], Ban Feng[3], Yingying Zhou[3,4], Qian Cheng[1], Yuan Li[1], Xingsheng Peng[1], Mengnan Wu[1], Lianshun Xie[3], Lei Xiao[1], Wenhao Zhou[1,2], Yuejun Chen[3, *], Man Xiong[1, *]

Supplemental Figures 1-7

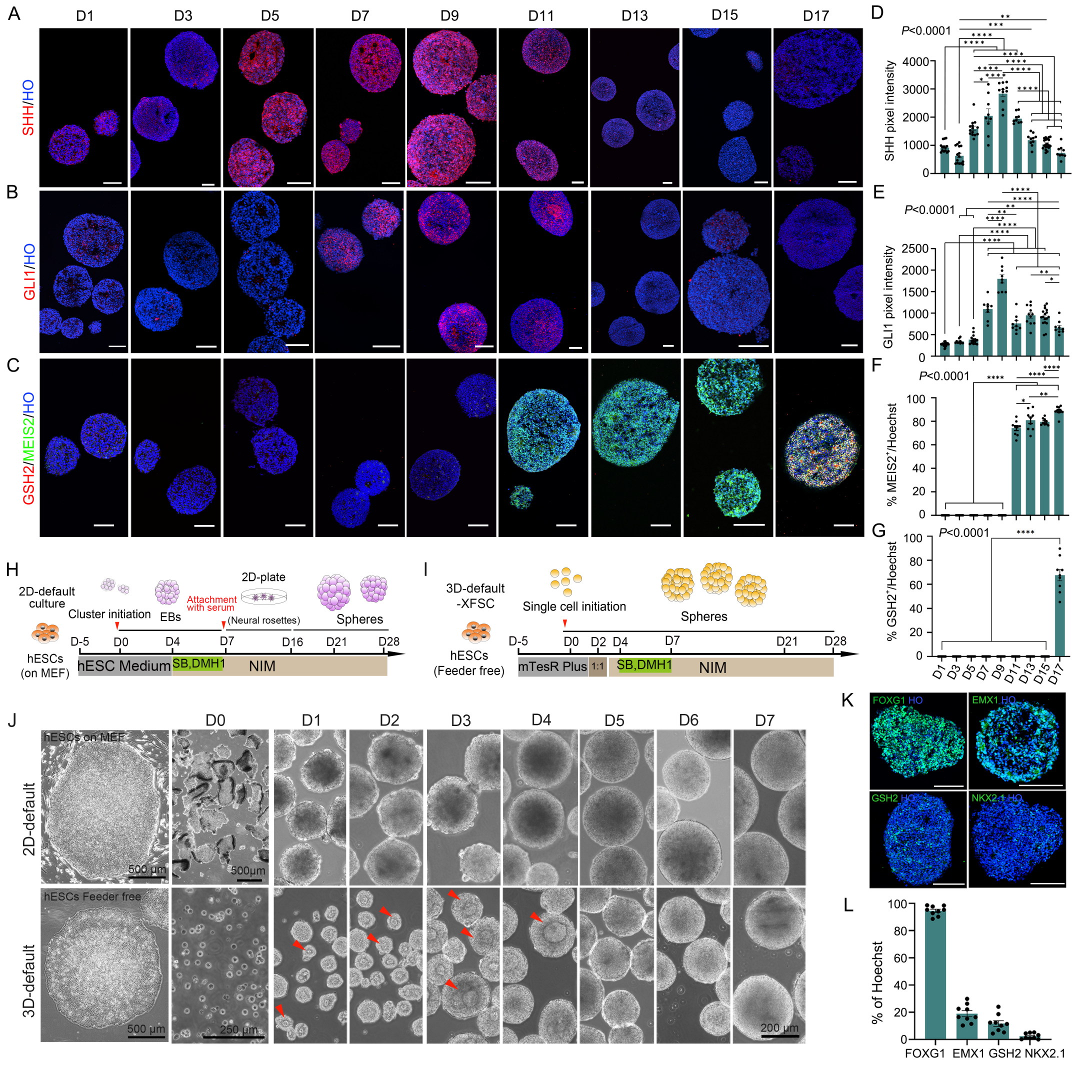

Supplemental Figure 1. Characterization of hESC-derived neurospheres differentiated by 3D-default XFSC and 2D-default EB culture. Related to Figure 1.

(**A-C**) The dynamic expression of SHH (A), GLI1 (B), GSH2 and MEIS2 (**C**) in spheres from day 1 to day 17 during neural differentiation by 3D-default XFSC. Scale bar: 100μm. (**D-E**) Quantitative analysis of SHH and GLI1 expression levels. (**F-G**) Quantification of MEIS2 and GSH2 immuno-positive cells to total. (**H-I**) Schematic diagram of 2D-default culture and 3D-default-XFSC neural differentiation methods. (**J**) Bright-field overview images of hESCs before neural differentiation and spheres during the first 7 days after neural differentiation. (**K-L**) Immunostaining and quantification of FOXG1, EMX1, GSH2 and NKX2.1 in day 20 neural progenitors differentiated by 2D-default culture. Scale bar: 100μm. Data are presented as the mean ± SEM. n = 3

biological replicates, One-way ANOVA followed by Tukey's multiple comparisons test. $*P < 0.05$, $**P < 0.01$, $***P < 0.001$, $****P < 0.0001$.

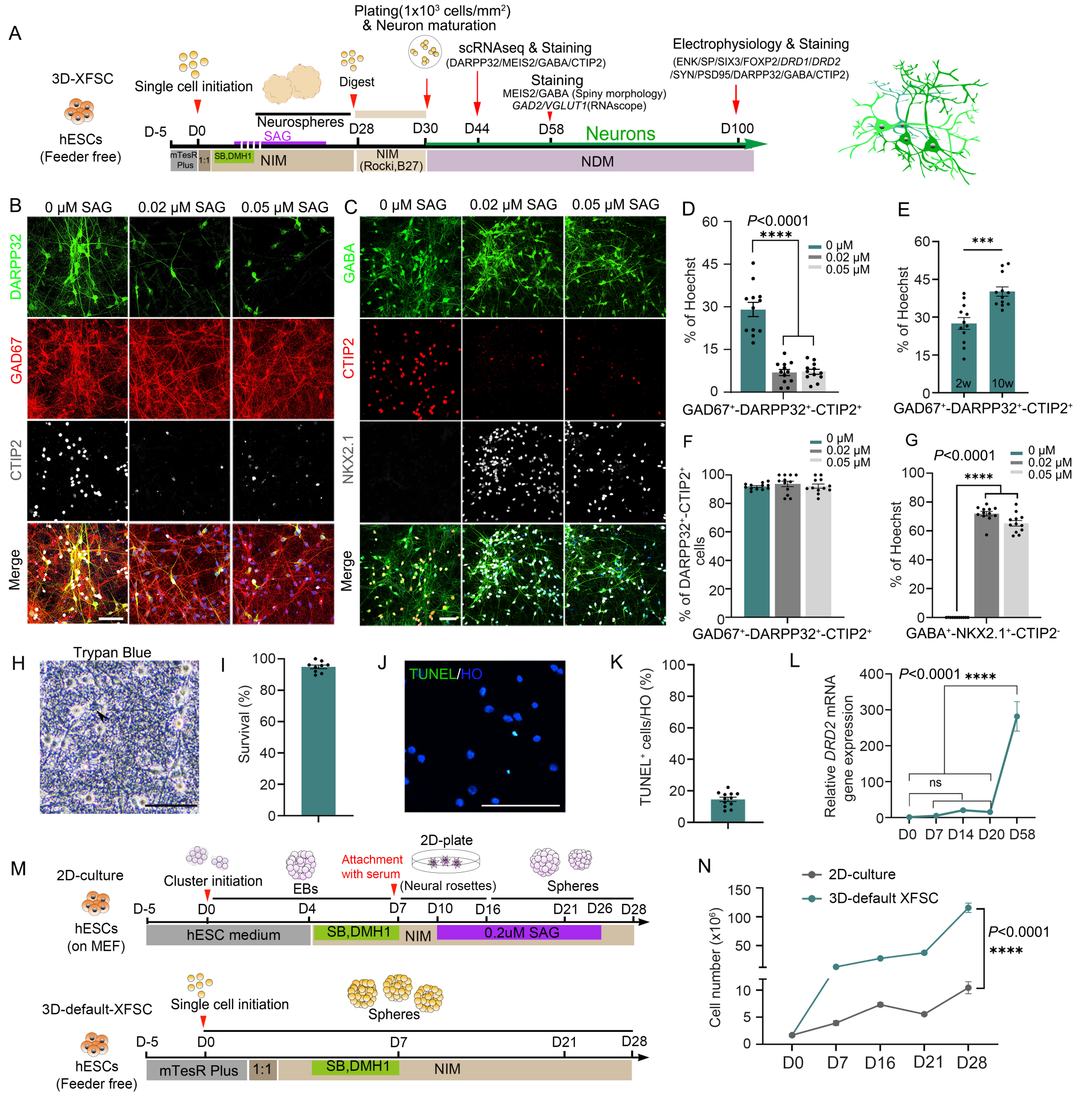

Supplemental Figure 2. Schematic diagrams for experimental procedures and characterization of neural progenitors and neurons differentiated by 3D-default XFSC. Related to Figure 2.

(**A**) Schematic diagram of differentiation protocol with time points for each experiment. (**B-C**) Immunostaining of DARPP32/GAD67/CTIP2 and GABA/CTIP2/NKX2.1 with 0, 0.02, 0.05 μM SAG treatment 2 weeks after neuron maturation. Scale bar: 50 μm. (**D** and **F**) Quantification of GAD67^{+}-DARPP32^{+}-CTIP2^{+} triple-labeled cells among total cells (**D**), or among DARPP32^{+}-CTIP2^{+} double-labeled cells. (**F**) Data were analyzed by One-way ANOVA followed by Tukey's multiple comparison test. (**E**) Quantification of GAD67^{+}-DARPP32^{+}-CTIP2^{+} triple-labeled cells among total in 3D-default XFSC-derived cells (without SAG) at 2 and 10 weeks after neuron maturation. Data were analyzed by Student's t-test. (**G**) Quantification of GABA^{+}-NKX2.1^{+}-CTIP2^{-} among total cells generated by 3D-default XFSC with 0, 0.02, 0.05 μm SAG treatment 2 weeks after neuron maturation. Data were analyzed by One-way ANOVA followed by Tukey's

multiple comparison test. (**H**-**I**) Representative images and quantification for the detection of live cells by Trypan Blue after 14 weeks culture. Scale bar: 100 μm. (**J**-**K**) Representative image and quantification for TUNEL staining for the detection of apoptotic cells after 14 weeks culture. Scale bar: 100 μm. (**L**) Relative mRNA expression of *DRD2* at different time point during 3D-default XFSC. Data were analyzed by One-way ANOVA followed by Tukey's multiple comparison test. (**M**-**N**) The neural differentiation protocols of 2D-culture for MSN and 3D-default XFSC (**M**), and the growth curve of striatal neuron differentiation from day 0 to day 28 (**N**). Data were analyzed by Two-way ANOVA. Data are presented as the mean ± SEM., n = 3 biological replicates, *$P < 0.05$, **$P < 0.01$, ***$P < 0.001$, ****$P < 0.0001$.

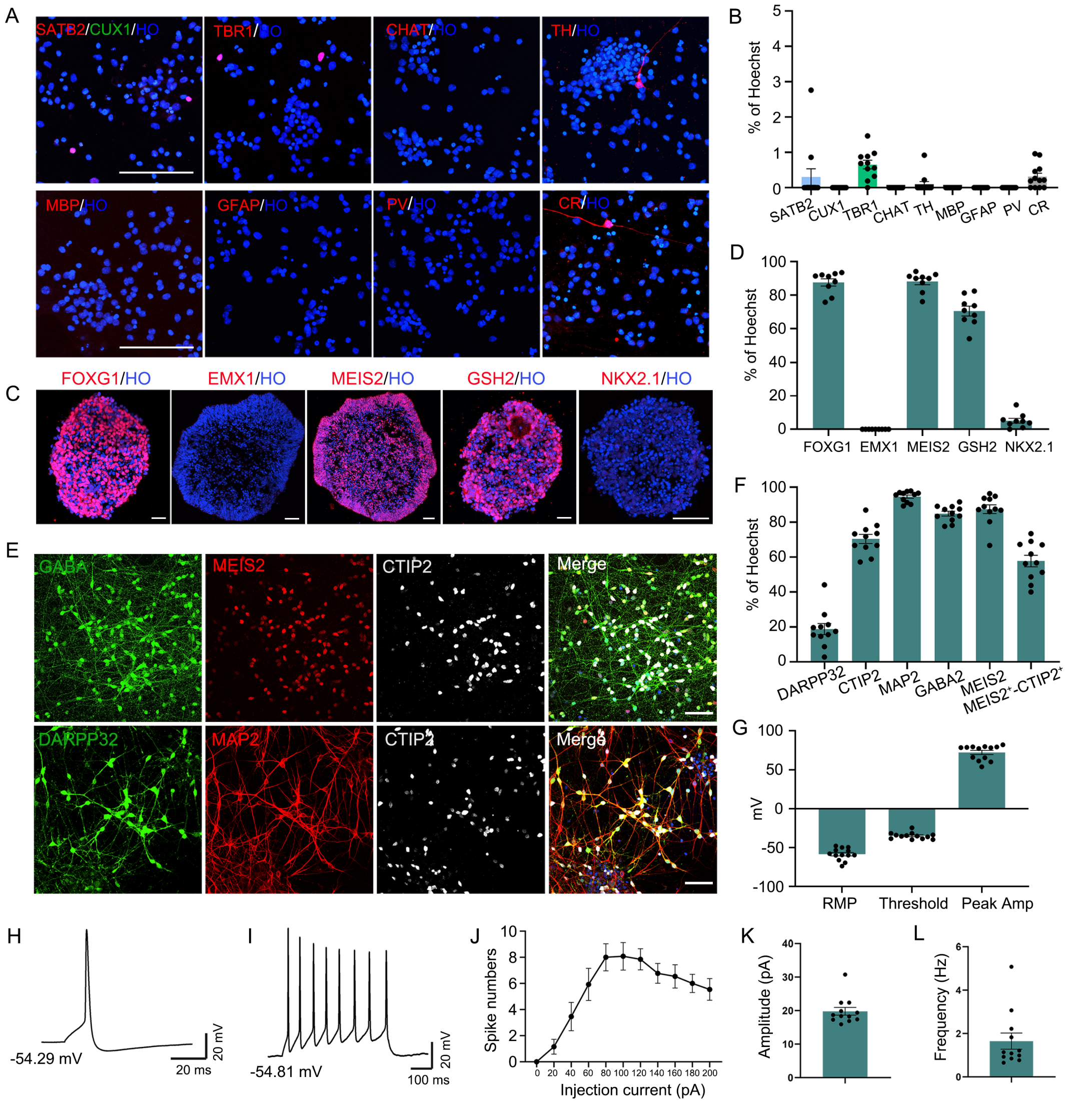

Supplemental Figure 3. Characterization of neurons derived from hESCs and iPSCs by 3D-default-XFSC in vitro. Related to Figure 2.

(**A-B**) Immunostaining and quantification for cortical neuron markers (SATB2, CUX1, TBR1), cholinergic neuron marker (CHAT), dopaminergic neuron marker (TH), oligodendrocyte marker (MBP), astrocyte marker (GFAP), interneuron marker (PV, CR) of cells at 4 weeks after neuron maturation. Scale bar: 100 μm. (**C-D**) Immunostaining and quantification for FOXG1, EMX1, MEIS2, GSH2 and NKX2.1 in day 20 neurospheres derived from iPSCs. Scale bar: 50 μm. (**E-F**) Immunostaining and quantification for DARPP32, CTIP2, MAP2, GABA, MEIS2 and MEIS2/CTIP2 in iPSCs-derived neurons after 2 weeks of maturation. Scale bar: 50 μm. (**G**) The quantification of resting membrane potentials (RMP), threshold, and peak amplitude (peak Amp) of current-induced action potentials (AP) in 3D-default XFSC-derived neurons after 8 weeks of in vitro maturation (n = 13 cells). Data are presented as the mean ± SEM. (**H-I**) Typical traces of current-induced single AP and multiple APs. (**J-L**) Statistics of spike numbers at different injection currents (**J**), the amplitude (**K**) and frequency (**L**) of sIPSCs in 3D-default XFSC-derived neurons after 8 weeks in vitro

maturation (n = 12 cells). Data are presented as the mean ± SEM. n = 3 biological replicates.

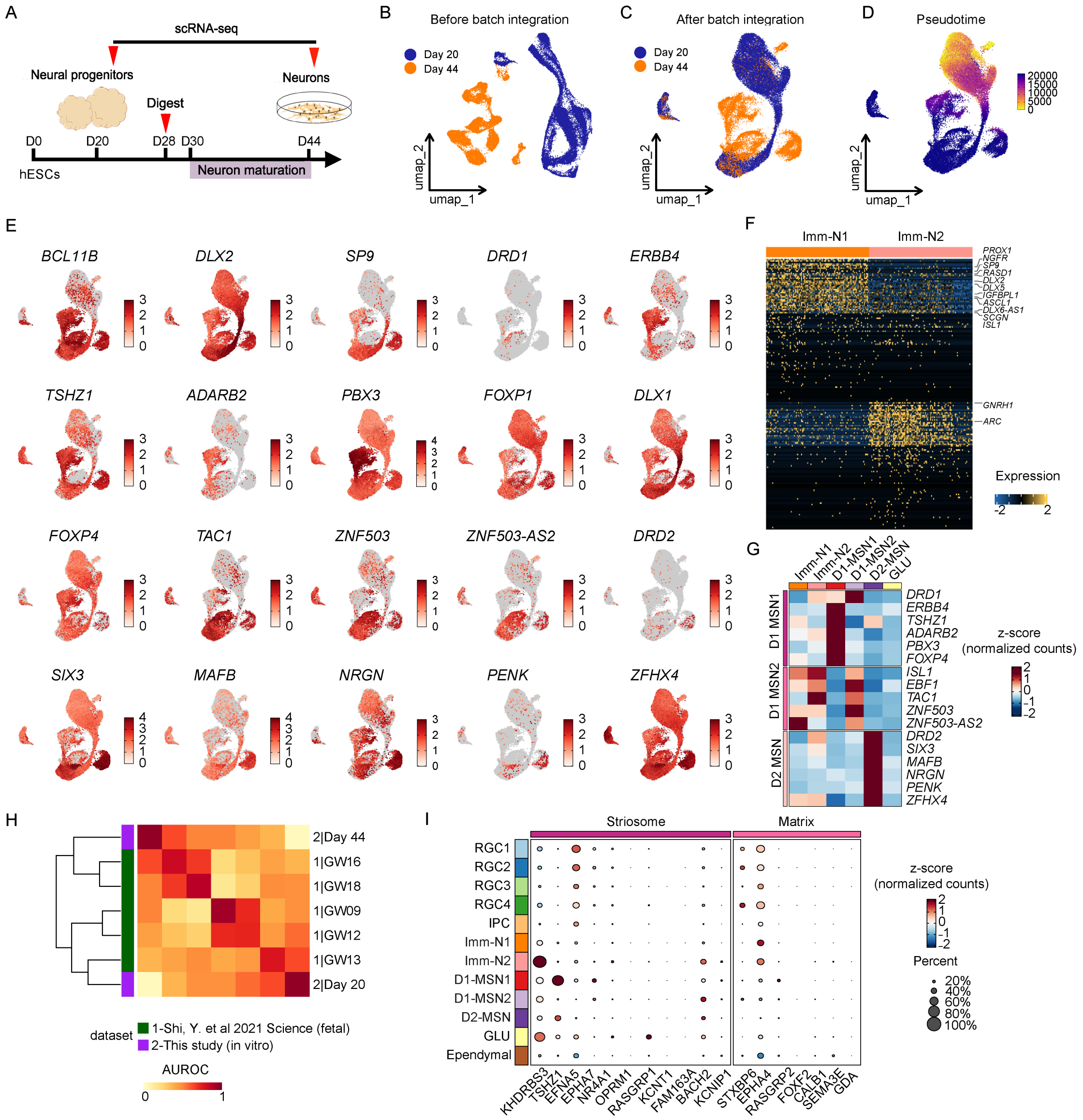

Supplemental Figure 4. Transcriptional features of neural progenitors and neurons generated by 3D-default XFSC. Related to Figure 3.

(**A**) Schematic diagram of single-cell RNA sequencing experiments conducted at different differentiation stages (day 20 and day 44, 2 replicates for each time point). (**B** and **C**) UMAP plot showing cells annotated by differentiation stage before and after batch integration. (**D**) UMAP visualization of pseudotime across all cells. (**E**) UMAP plots displaying the expression of well-known marker genes in radial glial cells, MGE, pan-LGE, D1-MSN and D2-MSN. (**F**) Heatmap illustrating DEGs enriched in Imm-N1 and Imm-N2. (**G**) Heatmap showing the expression of D1- and D2-MSN subtype-specific marker genes across neuronal clusters. (**H**) Heatmap showing pairwise transcriptional correlations between 3D-default XFSC-derived day 20 and day 44 cells in vitro and their corresponding populations (LGE and GE progenitors) in the developing human brain dataset (1). (**I**) Dot plot showing the expression of marker

genes associated with striosome and matrix compartments in cells generated by 3D-default-XFSC.

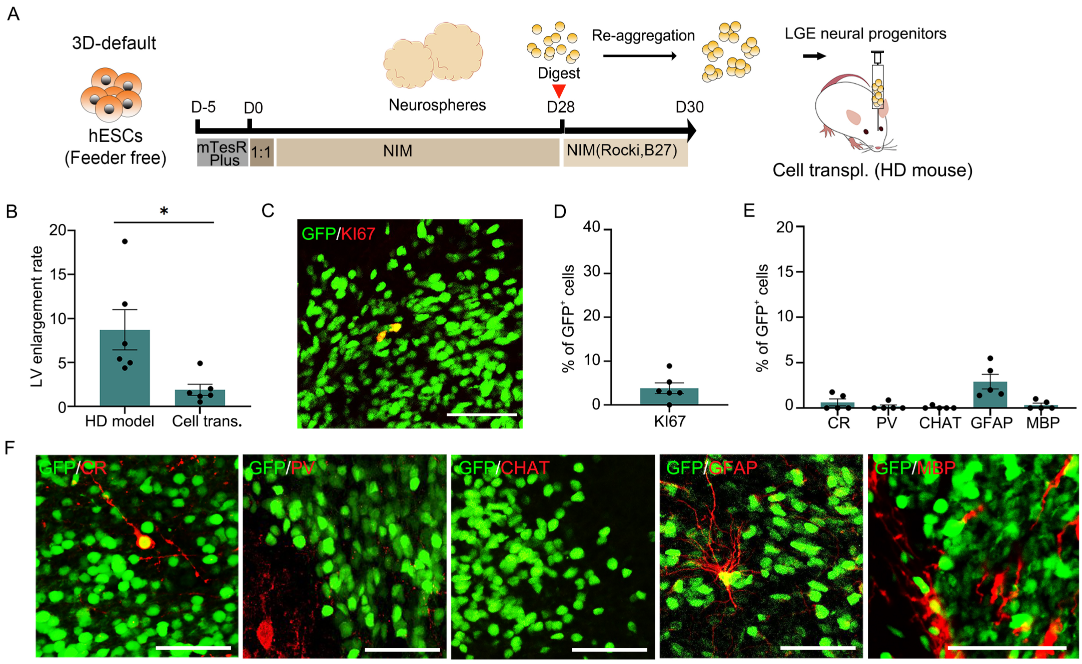

Supplemental Figure 5. Characterization of cellular composition in graft at 5 months after transplantation. Related to Figure 4.

(**A**) Schematic diagram showing the protocol for neural differentiation and cells transplantation. (**B**) Quantification of ipsi-lateral ventricular (LV) areas in HD model mice with cell transplantation, compared to untreated HD controls. (**C**-**D**) Representative immunofluorescent image and the quantification of GFP and KI67 immunopositive cells in the graft 2MPT in HD model mice. (**E**-**F**) Representative immunostaining images and quantification for GFP, CR, PV, CHAT, GFAP and MBP in the graft 5MPT in HD model mice. Scale bar: 50μm. n=5 mice. Data were analyzed by Student's t-test and presented as the mean ± SEM. **P* < 0.05.

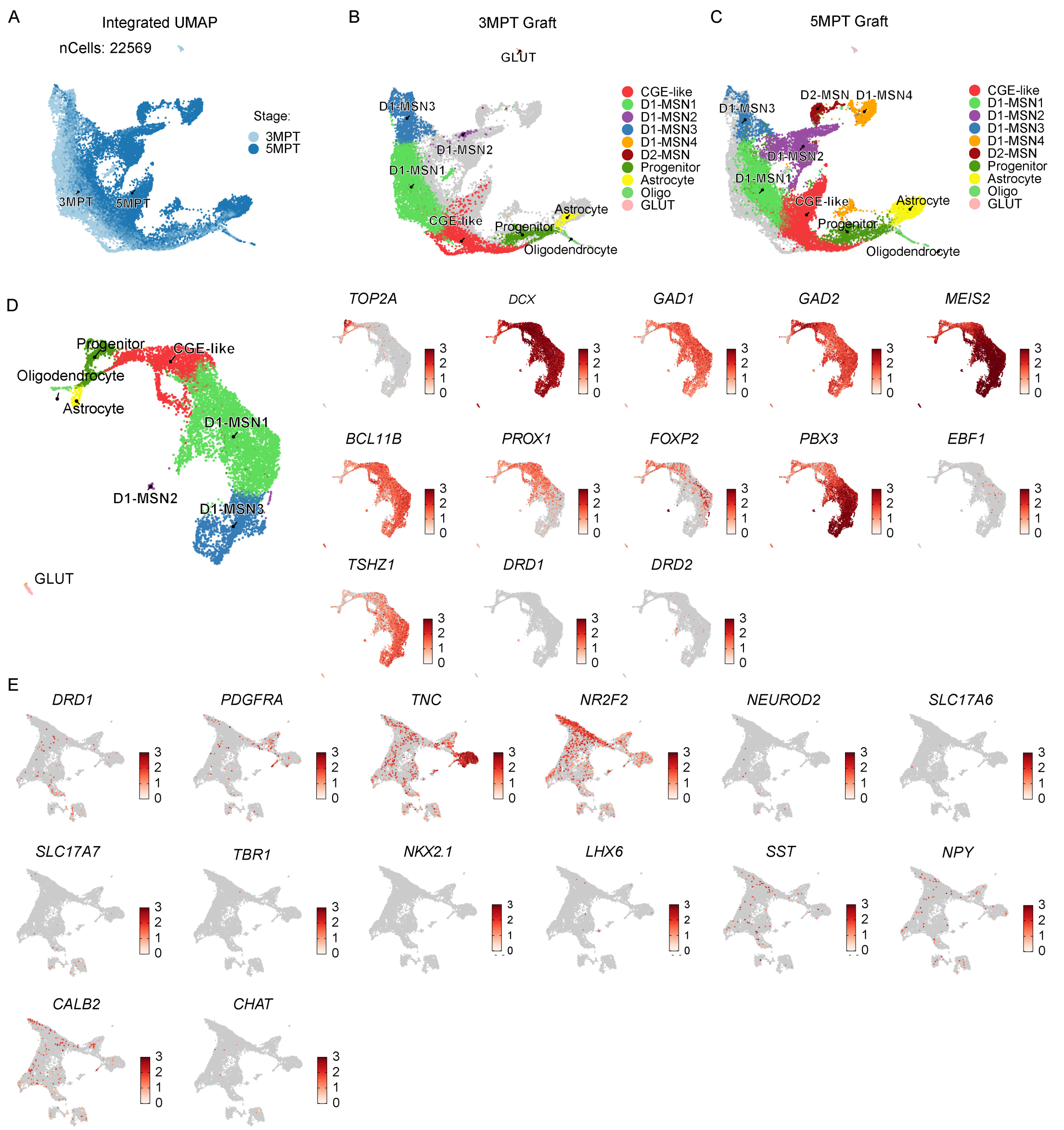

Supplemental Figure 6. Transcriptomic characteristics of grafts at different time points post-transplantation. Related to Figure 5.

(**A**) UMAP visualization of integrated datasets, with cells annotated by time point (3MPT and 5MPT). (**B-C**) UMAP visualization of integrated datasets annotated by cell clusters from grafts at different time points (3MPT and 5MPT). (**D**) UMAP visualization of human cell clusters in the 3 MPT graft and the representative marker genes for different cell clusters. (**E**) UMAP plots showing the expression of marker genes in the 5MPT graft, including markers for D1-MSN (*DRD1*), oligodendrocyte (*PDGFRA*), astrocyte (*TNC*), CGE marker genes (*NR2F2*), glutamatergic neuron marker genes (*NEUROD2, SLC17A6, SLC17A7, TBR1*), MGE marker genes (*NKX2-1, LHX6*), and interneuron marker genes (*SST, NPY, CALB2, CHAT*) in 5 months graft.

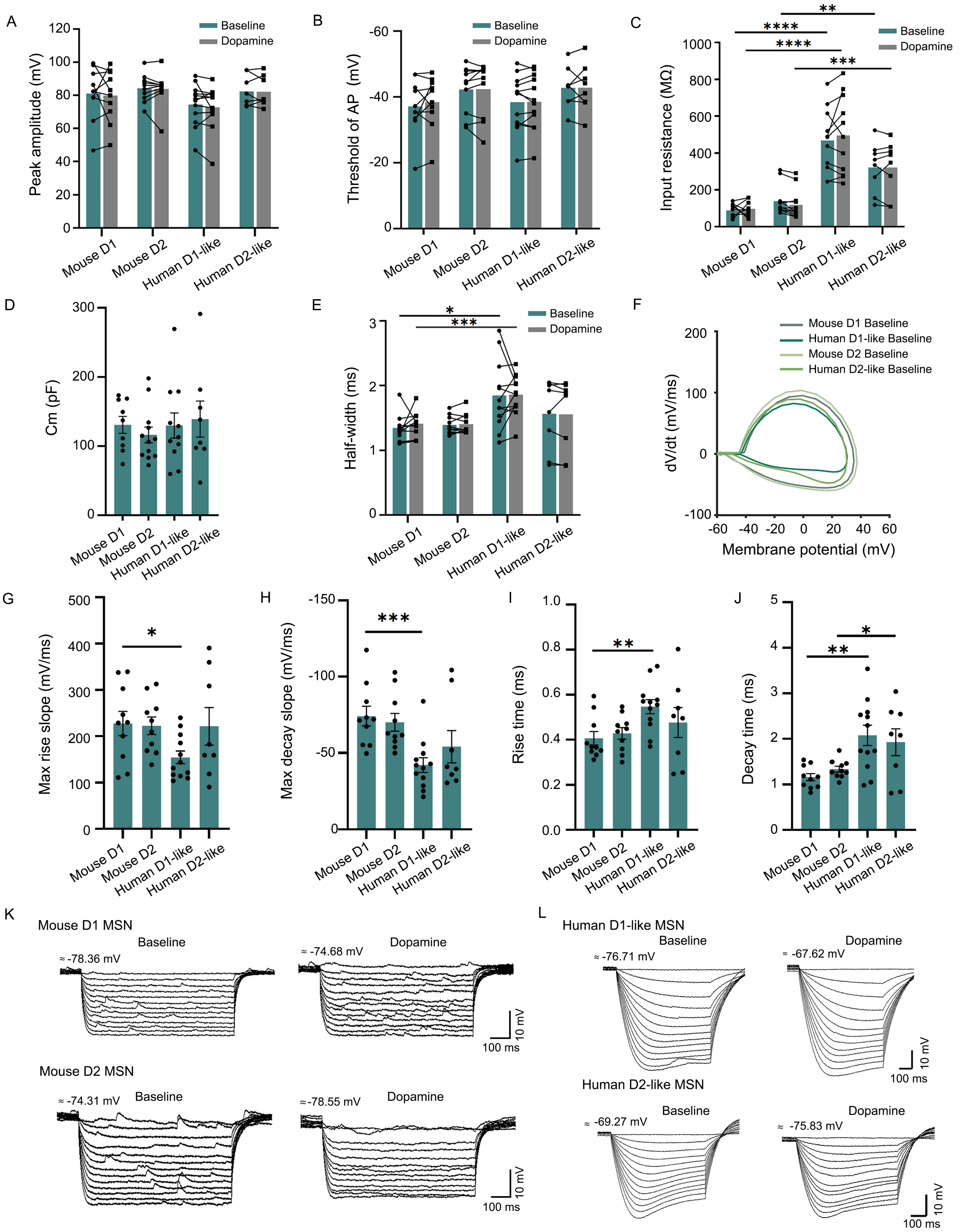

Supplemental Figure 7. Electrophysiological properties of mouse D1-/D2-MSNs and human D1-like/D2-like MSNs in the graft. Related to Figure 6.

(**A-B**) Comparison of peak amplitude (**A**) and AP threshold (**B**) in mouse D1-/D2-MSNs and human D1-like/D2-like MSNs before and after dopamine administration. (**C**) Comparison of input resistance in mouse D1-/D2-MSNs and human D1-like/D2-like MSNs before and after dopamine administration. (**D**) Comparison of membrane capacitance in mouse D1-/D2-MSN and transplanted human D1-like/D2-like MSNs. (**E**) Comparison of AP half-width in mouse D1-/D2-MSNs and human D1-like/D2-like

MSNs under baseline and dopamine conditions. (**F**) Representative AP phase plots show the rate of change of membrane potential (mV/ms) for mouse D1-/D2-MSNs and transplanted human D1-like/D2-like MSNs. (**G-J**) Maximum rise slope of action potentials (G), maximum decay slope of action potentials (**H**), spike rise time, defined as the time between 10% and 90% of spike amplitude during the depolarizing phase (**I**), spike decay time, defined as the time between 90% and 10% of spike amplitude during the repolarizing phase (**J**) in mouse D1-/D2-MSNs and human D1-like/D2-like MSNs. (**K**-**L**) Voltage response of mouse D1-/D2-MSNs (**K**) and human D1-like/D2-like MSNs (**L**) to negative current pulses (-120 pA to 0 pA, step 10 pA, duration 500 ms) before and after dopamine administration. n=10 cells from 5 mice for mouse D1 MSN; n=10-12 cells from 4 mice for mouse D2 MSN; n=11-12 cells from 6 mice for human D1-like and n=8 cells from 6 mice for human D2-like MSN. Paired Student's t test was used between the group before and after dopamine administration. The statistical differences between mouse and human were determined by unpaired Student's t tests. Data are presented as the mean ± SEM. $^{*}P < 0.05$, $^{**}P < 0.01$, $^{***}P < 0.001$, $^{****}P < 0.0001$.

Supplemental Methods

hPSCs maintenance and passage

Human H9 ESCs [line WA09 (WiCell), passages 20–40, WiCell Agreement No. 16-W0060] or iPSCs were maintained under feeder free conditions with mTeSR Plus (STEMCELL Technologies) on the Vitronectin (Life Technologies)-coated six-well plate. The stem cells were cultured and replenished with fresh medium daily in a 37°C humidified incubator with 5% CO_2. hPSCs were digested into single cell for passage using TrypLE Express Enzyme (Life Technologies) when colonies became 80% confluent.

Generation of hiPSCs from blood mononuclear cells

The hiPSC line was generated by the Pluripotent Stem Cell Core Facility at the CAS Center for Excellence in Brain Science and Intelligence Technology, with ethical approval from the Ethics Committee of the Institute of Neuroscience, CAS Center for Excellence in Brain Science and Intelligence Technology (Approval No. CEBSIT-2021033). The process of iPSCs reprogramming was similar as protocols previously described (2).

2D and 3D cell culture

For 2D-default neural differentiation (3, 4). hESCs are cultured on MEF with hESC medium consisting of DMEM/F-12, 1 × GlutaMAX, 1 × non-essential amino acids and 0.1 mM β-mercaptoethanol. After five days of culture, the hESCs are digested into

bigger cell clusters by dispase to initiate neural differentiation. For the first three days, the medium used is the same as that for culturing hESCs on MEF. For 3D-default culture, the starting hESCs are cultured with feeder free system. After five days of culture, the hESCs were dissociated into single cells using TrypLE Express Enzyme to initiate the neural differentiation with mTesR Plus for the first day, 1:1 mTesR Plus: neural induction medium (NIM) (DMEM/F-12 (Gibco), 1 × MEM Non-Essential Amino Acids (NEAA, Gibco), 1 × N2 supplement (Gibco) for the second day. Then, both 2D- and 3D-culture systems used NIM as the culture medium. For 2D-default culture, From Day 4 to Day 6, change medium into NIM with 2 μM TGF-β inhibitors SB431542 (Stemgent) and 2 μM BMP inhibitor DMH-1 (Tocris). On Day 7, spheres were attached to six-well plates in NIM containing 5% fetal bovine serum to form neural rosettes. Then the medium was changed with NIM next day. On Day 15 or 16, the neural rosettes on six-well plates were blown off and cultured with NIM containing 1 × B27 and 0.5 μM ROCK inhibitor in T25 flasks. Then the medium was changed with NIM every two days until day 26. 2D-culture for MSNs was performed following previous reports (5, 6). The procedure was similar with the 2D-default culture, except for adding 0.2 μM SAG from Day 10 to Day 26. For 3D-XFSC, from day 2, the spheres were suspension and cultured with NIM until neuron maturation. From day 3 to day 7, 2 μM SB431542 and 2 μM DMH-1 were added to induce neuroepithelia identity. In order to explore the proper concentration of SAG, 0, 0.02, and 0.05 μM smoothened agonist SAG (Millipore) were added from day 5 to day 20 during neural differentiation. Neurospheres were differentiated for 28 days with varying concentrations of SAG (0,

0.02 or 0.05 μM) from day 5 to day 20, followed by dissociation using accutase at 37℃ for 6 minutes to obtain very small cell clusters (2-3 cells). These cell clusters were cultured in NIM supplemented with ROCK inhibitor (reduce cell death) and B27 (provide nutrients) for 2 days. Subsequently, the re-aggregated small neurospheres were collected and seeded onto Matrigel-coated coverslips at a density of 1000 cells/mm^2, and cultured in neuronal maturation medium (NDM) containing 1 × MEM NEAA, 1 × N2 supplement, 1 × B27 supplement, 200 μM ascorbic acid (AA, Sigma), 10 ng/mL glial-derived neurotrophic factor (GDNF, PeproTech), 10 ng/mL brain-derived neurotrophic factor (BDNF, PeproTech), 1 μM cylic adenosine monophosphate (cAMP, Millipore), 10 ng/mL insulin-like growth factor 1 (IGF1, Peprotech), and 0.2 μM Compound E (Millipore). Two weeks post-maturation, cells were stained for DARPP32/MEIS2/GABA/CTIP2 to analyze striatal neuron identity and differentiation efficiency or undergo scRNA-seq. Cells were stained for MEIS2/GABA and GAD2/VGLUT1 at 4 weeks post-maturation. Electrophysiology recording and immunofluorescent staining for ENK/SP/SIX3/ FOXP2/DRD1/DRD2/ SYN/PSD95 and GABA/DARPP32/CTIP2 were performed 10 weeks post-maturation. To assess the capacity of long-term maintenance of striatal neural progenitor cell fate, we followed a similar procedure as described above for cell dissociation and maturation. However, in this experiment, neurospheres were cultured for varying durations (14, 21, 28, 35, and 54 days) before maturation for 2 weeks. Finally, cells were stained for DARPP32/MEIS2/GABA/CTIP2 to assess striatal neuron identity.

Large-scale expansion of neurospheres

The neurospheres derived from 3D-default XFSC (day 16) were digested and plated on 96-well plates at the density of 10,000 cells/well and refreshed with the NIM every other day. These aggregates were dissociated using TrypLE™ Express Enzyme and counted by Countess™ 3 FL Automated Cell Counter (Invitrogen) at day 21, 28, 35, 56 during neural differentiation, respectively. For comparative experiment between 2D and 3D culture for MSNs, we initiate neural differentiation with same number of stem cells and digest neurospheres to counted cells number after 7, 16, 21, 28 days of differentiation.

Quantitative real-time polymerase chain reaction (qRT-PCR)

RNA samples were obtained from cultured human cells by TRIzol reagent (Ambion). Prime Script™ RT Master Mix (Takara) were used for reverse transcription reaction. Real-time PCR (RT-PCR) was performed by using TB Green Premix Ex Taq (Takara) with Applied Biosystems 7500 Real-Time PCR System. The expression levels of the target genes were normalized to the housekeeping gene *GAPDH*, with the cell samples from day 0 during neural differentiation utilized as the negative control.

Western blots

Protein was isolated using RIPA lysis buffer (Epizyme Biotech) containing 1 × proteinase inhibitor cocktail tablets (Roche Diagnostics GmbH). The concentration of protein was calculated using a BCA protein assay kit (Biosharp). For immunoblotting,

samples were mixed with 5 × loading buffer (Epizyme Biotech), boiled for 5 min at 100°C. 20 μg protein samples were separated on a 10% polyacrylamide gel (Epizyme Biotech). The gel was subjected to electrophoresis at 80 V for 30 min, then at 110 V for 90 min. Proteins were then transferred onto a PVDF membrane (Cytiva) at 250 mA for 105 min. The membrane was blocked with 5% non-fat milk in Tris-buffered saline with 0.1% Tween-20 (TBST, Biosharp) for 1 hour. The membrane was washed with TBST twice and then incubated with primary antibody diluted in blocking buffer overnight at 4°C (anti-GAPDH was diluted 1:5000; anti-SHH was diluted 1:500). The membrane was washed with TBST three times and incubated with secondary antibody in TBST for 1 hour with shaking. After washing with TBST for three times, protein bands were detected with the chemiluminescence method using Super-sensitive ECL chemiluminescent substrate (Biosharp).

HD model mouse and Cell Transplantation

The HD mouse model was conducted as described previously(5). Briefly, severe combined immunodeficiency (SCID beige) mice (6-8 weeks) were injected 1 μL of 0.2 M Quinolinic Acid (QA, Sigma) into the right striatum (anterior-posterior [AP]: +0.8 mm; medial-lateral [ML]: +1.7 mm; dorsal-ventral [DV]: −3.5 mm, from skull). Neurospheres generated by 3D-default XFSC at day 28 were digested by Accutase for 6 minutes and suspended with NIM in the presence of 0.5 μM ROCK inhibitor and 1 × B27 Supplement without Vitamin A (Gibco) for 1-2 days. Then, the small cell clusters were collected and resuspended in artificial cerebrospinal fluid (ACSF) containing

BDNF (20 ng/ml), ROCK inhibitor (1 μM) and 1 × B27. Adult SCID mice were anesthetized with isoflurane mixed in air and about 1 × 10^5 cells were injected into the ipsilateral striatum on a coordinator ([AP]: +0.7 mm, [ML]: +1.8 mm, [DV]: −3.5 mm, from skull). The HD model mice were randomly grouped and received cell transplantation or ACSF (negative control) 3-4 weeks after QA lesion.

Immunofluorescent staining

The 3D neurospheres dehydrated by 30% sucrose solution for 24 h and cut 16 μm thick sections, meanwhile collecting sections on adhesive slides. Neurospheres from different developmental stages or neurons cultured on the coverslips were fixed with 4% paraformaldehyde (PFA) for 15 min at room temperature. Subsequently, they were washed with DPBS three times at 10-minute intervals. The cell samples were then permeabilized with 0.2% Triton X-100 in DPBS for 15 min, followed by incubation in 10% donkey serum for 1 h at room temperature. Then, the cell samples were incubated with primary antibodies, including GABA, MEIS2, CTIP2, DARPP32, GSH2, ASCL1, SHH, GLI1, SP, ENK, NKX2.1, etc. (detailed antibody information was provided in Supplemental Table 5), at 4°C overnight. Sections were incubated with secondary antibodies in 5% donkey serum for 1 h at room temperature. Nuclei were labeled with Hoechst 33342 (Sigma). Sections were mounted using fluorescence mounting medium (Southern Biotech). For staining mouse brains, the brain slices were treated with blocking solution (10% donkey serum and 0.3% Triton) for 1 h before incubated with primary antibodies for 1-3 nights. To detect the survival rate of cells after 10 weeks

maturation, the coverslips were incubated with Trypan Blue for 1 min at room temperature. Detection of live cells was assessed in bright field. For TUNEL staining, One Step TUNEL Apoptosis Assay Kit (Beyotime, China) was used according to manufacturer's instructions. Coverslips were fixed by 4% PFA and then washed with PBS three times. Next, the samples were permeabilized with 0.2% Triton X-100 for 10 min. After washing with PBS three times, the samples were incubated in the TUNEL mixture (1:20 dilution of terminal deoxynucleotidyl transferase in label solution) in a humid chamber for 45 min at 37°C. After TUNEL labelling, nuclei were stained with Hoechst 33342.

Tyramide signal amplification (TSA)

The brains were fixed in 4% PFA, sectioned coronally at a thickness of 30 μm. Coverslips were fixed by 4% PFA for 15 min. Next, washed with PBS three times. Endogenous peroxidase activity was blocked by incubating the sections or coverslips in 3% hydrogen peroxide solution (Thermo Scientific) for 1 h at room temperature. After brief PBS washes, samples were incubated in blocking solution (0.6% triton in 10% goat serum) for 1 h, and then label the cells with primary antibody (Rabbit anti-GABA antibody) at 4°C overnight. After washing 3 times for 5 min in PBS, the samples were incubated with SuperBoost Goat anti-Rabbit Poly HRP (Thermo Scientific) for 1 h at room temperature, washed 3 times with PBS and then subjected to signal amplification using Alexa Fluor 647 Tyramide (Thermo Scientific) for 8 min. Following washed in Reaction Stop Reagent (Thermo Scientific) for 5 min, the samples

were subjected to boiling citrate buffer for 15 min. After washed with PBS three times, the slices were subjected to staining using rabbit anti-CTIP2 antibody or rabbit anti-DARPP32 antibody, employing the same staining procedure as described for GABA staining but using other Alexa Fluor Tyramide (Thermo Scientific). After TSA labeling, the brain slices were stained with mouse anti-human Nuclei (hN) antibody with procedure described in part of immunofluorescent staining. Images were taken on Leica SP8 or Nikon NSPARC microscope.

RNAscope

The samples from in vitro or in vivo were fixed with 4% PFA prepared with diethyl pyrocarbonate (DEPC) water for 15 min. Then, the samples were treated by hydrogen peroxide (Advanced Cell Diagnostics, ACD) for 20 min at room temperature. After washing, the samples were placed in RNAscope Target Retrieval Reagents (ACD) for 6 min at 97 °C. Next, they were rinsed in 100% alcohol for 3 min and exposed to protease III (ACD) for 20 min at 40°C. Then, the samples were incubated with probes for 2 h at 40°C. Following hybridization with AMP 1, 2, and 3, they were treated with each corresponding secondary antibody. Images were acquired by the Nikon TIE A1 plus confocal microscope or Nikon AX confocal microscope.

Single cell sequencing and data analysis

We employed scRNA-seq for in vitro samples to obtain a comprehensive view of gene expression within intact cells. In contrast, for the in vivo graft, we utilized scRNA-seq

to retain more neurons in such samples. For in vitro cells: neurospheres (day 20) or neurons (day 44) were digested using TrypLE at 37°C for 10-15 minutes, followed by three washes with NIM and centrifugation at 400 × g for 3 minutes. The resulting cell suspension was passed through a 35 μm cell strainer (BD Biosciences) to obtain the single-cell suspension for sequencing. Library preparation was performed using the Chromium Single Cell 3' Reagent Kits v3 (10 × Genomics), according to the manufacturer's standard protocol. Libraries were constructed by Shanghai Biochip Co., Ltd. and sequenced on an Illumina NovaSeq platform in paired-end mode (PE150), with a read length of 150 base pairs.

scRNA-seq data analysis of cultured cells:

1. Quality control and filtering: Raw sequencing data were first processed using the Cellranger pipeline (v7.0.1, 10 × Genomics) with GRCh38-2020-A as the reference. The resulting gene expression matrices were imported into Seurat (v4.3.0) for further analysis. Low-quality cells were filtered out based on the following criteria: cells with fewer than 500 genes or more than 8,000 genes, fewer than 1,000 counts or more than 30,000 counts, and cells with mitochondrial gene content exceeding 8%. Doublets were detected using DoubletFinder (v2.0.3), and the filtering ratio was determined based on the number of cells recovered.

2. Integration and clustering: After initial quality control, the top 4,000 highly variable genes were identified using the "FindVariableFeatures" function. Shared features across datasets were identified using the "SelectIntegrationFeatures" function, and integration anchors were determined using the "FindIntegrationAnchors" function. The datasets

were then integrated using the “IntegrateData” function to create a unified, batch-corrected expression matrix for downstream analysis. The clustering results were visualized using UMAP.

3. Differential gene expression analysis: DEGs between clusters and specific cell types were identified using the “FindAllMarkers” function in Seurat. Pathway enrichment analysis was performed using KEGG or GO to identify key pathways involved in neuronal differentiation and cell lineage specification. For pseudotime analysis, the destiny (v3.12.0) package was used to order cells along differentiation trajectories based on gene expression changes.

4. Comparison with reference datasets: To compare the in vitro-derived cells with in vivo datasets, we integrated our scRNA-seq data with publicly available human datasets (GSE135827) in Seurat. Cell type annotations and correlation analyses were performed to assess the transcriptional similarity between *in vitro* and *in vivo* cell populations. Cluster-level similarity and developmental stage alignment were evaluated using MetaNeighbor (v1.18.0). In addition, the integrated dataset was spatially projected onto embryonic brain maps using VoxHunt (v1.0.1), based on E13 regional marker genes from the Allen Developing Mouse Brain Atlas.

For in vivo snRNA-seq, graft tissues from the striatum were dissected under a fluorescence stereomicroscope. The tissue was then gently homogenized using a tissue grinder. After confirming sufficient dissociation under a microscope, the suspension was filtered through 70 and 40 μm cell strainers. The grinder was rinsed thoroughly, and the filtrate was collected into a centrifuge tube. Nuclei were pelleted by

centrifugation and resuspended for downstream snRNA-seq library preparation. All graft library preparation was performed using the Chromium Single Cell 3'Reagent Kits v3 (10 × Genomics), according to the manufacturer's standard protocol. Libraries were constructed by Shanghai Biochip Co.,Ltd. and sequenced on an Illumina NovaSeq platform in paired-end mode (PE150), with a read length of 150 base pairs.

Data analysis of snRNA-seq:

1. Alignment and Quality Control: Raw sequencing data were processed using the Cellranger pipeline (v7.0.1, 10 × Genomics) in intron mode with the GRCh38_and_mm10-2020-A reference genome.

2. Mapping Statistics: 94.9% of reads were mapped to the genome, with 85.9% mapping to GRCh38 and 9.1% to mm10.

3. Nuclei Counts: A total of 16,352 nuclei were detected, comprising 15,354 human nuclei (GRCh38) and 1,282 mouse nuclei (mm10). The resulting gene expression matrices were imported into Seurat (v4.3.0) for further analysis. To filter out mouse-derived nuclei, we retained nuclei where over 50% of total counts were attributed to human genes, resulting in a final count of 15,168 human nuclei. For these nuclei, only genes with the GRCh38 prefix (28,080 human genes) were retained for downstream analysis.

4. Dimensionality Reduction and Clustering: Highly variable genes (top 3,000) were identified using the "FindVariableFeatures"function in Seurat. Principal component analysis (PCA) was run with 100 dimensions, followed by UMAP embedding, neighbor finding (50 dimensions), and clustering (resolution= 0.4). Low-quality clusters with

extremely higher or lower average nFeature_RNA and nCount_RNA compared to other clusters were further filtered. The final reclustered results were then visualized using UMAP.

5. Differential Gene Expression and Reference Dataset Comparison: These analyses were performed following the same methods used for the in vitro scRNA-seq data.

Whole-Cell Patch-Clamp Recording

Brain slices for electrophysiological recordings were prepared following a previously published protocol (6). Mice were deeply anesthetized with isoflurane and sacrificed. The brain was dissected rapidly and placed into oxygenated (95% O_2 and 5% CO_2) ice-cold ACSF, containing 252 mM Sucrose, 2.5 mM KCl, 0.5 mM $CaCl_2$, 6mM $MgSO_4$, 25 mM $NaHCO_3$, 1 mM NaH_2PO_4, and 10 mM glucose. Coronal brain slices (300 μm) at the level of the striatum were prepared using vibratome and then transferred into an incubating bath containing oxygenated ACSF (with 124 mM NaCl, 2.5 mM KCl, 2 mM $CaCl_2$, 2 mM $MgSO_4$, 25 mM $NaHCO_3$, 1 mM NaH_2PO_4, and 37 mM glucose) at room temperature. After 1 hour of incubation, brain slices were transferred to a recording chamber for visualizing through an Olympus microscope.

During whole-cell patch-clamp recordings, mouse D1- or D2-MSN or transplanted human neurons were identified by GFP, mCherry or tdTomato fluorescence. For AP recording, the patch pipettes filled with the solution containing 120 mM K-gluconate, 5 mM NaCl, 1 mM $MgCl_2$, 0.2 mM EGTA, 10 mM HEPES, 2 mM Mg-ATP, 0.1 mM Na_3-GTP and 10 mM phosphocreatine disodium. We used current (-40 - +320 pA, step

20 pA, duration 400 ms) to induce APs, and used step currents (20 ms, step 15 pA) to induce single AP. A pre-recording offset correction was performed using the Axon 700B amplifier prior to seal formation, without compensating for the liquid junction potential (LJP). To record sIPSCs and sEPSCs for brain slices, pipettes filled with the solution containing 112 mM $CsMeSO_3$, 5 mM TEA-Cl, 3.7 mM NaCl, 0.2 mM EGTA, 10 mM HEPES, 2 mM MgATP, 0.3 mM Na_3GTP and 5 mM QX-314. sEPSCs and sIPSCs were recorded in voltage-clamp mode holding at -70 mV and +10 mV, which was blocked by AMPA receptor antagonist CNQX or $GABA_A$ receptor antagonist PTX, respectively. Since sEPSCs and sIPSCs recordings were performed on the same cell, we included QX-314 in the internal solution to eliminate any potential interference from sodium currents generated at the depolarized potential (+10 mV) used for sIPSCs recordings. The initial access resistance was 15-30 MΩ, and monitored throughout the experiment. Whole-cell patch-clamp recordings in vitro were performed on coverslips placed in a chamber perfused with oxygenated ACSF. AP numbers were detected in response to equal amplitude current steps (0-200 pA, step 20 pA, duration 500 ms). The sIPSCs for in vitro cultured neurons were recorded at +10 mV. The solution filled in pipettes during AP or sIPSCs recording was the same as that used for brain slice recordings.

The effects of dopamine neuromodulation on mouse MSNs and grafted neurons were performed as descried before (7). Briefly, PTX (100 μM), APV (50 μM), CNQX (25 μM) were added to block the activation of $GABA_A$, AMPA, and APV receptors respectively. Dopamine (60 μM) was added into ACSF containing Ascorbate (1 mM) and the currents were recorded 3 min later. For optogenetic experiment, we inject AAV-

mCamKIIa-hChR2-EGFP virus into 3 positions within cortex M1 area ([AP]:+1.9 mm, [ML]:+1.5 mm, [DV]:−0.7 mm; [AP]:+1.35 mm, [ML]:+1.5 mm, [DV]:−0.7 mm; [AP]:+0.8 mm, [ML]:+1.5 mm, [DV]:−0.7 mm) or inject AAV-mCamKIIa-Cre and AAV-FLEX-ChrimsonR-tdTomato virus into thalamus ([AP]: -2.2 mm, [ML]:+0.7 mm, [DV]:−3.2 mm). Three weeks later, we recorded induced AP of the infected host cells and induced EPSCs of grafted cells after light stimulation (470 nm or 635 nm for 2 ms, 1.5 mW/mm^2). In all cases, biocytin (0.5%) was introduced into the recording solution to identify the morphological properties of the recorded neurons.

Behavioral tests

To evaluate motor behaviors, animals were conducted behavioral tests at 0-(pre-transplantation and 2 weeks post HD modeling), 2- and 5-months post cell transplantation following protocols descried before (6). Animals of HD + ASCF and WT group (healthy mice) were conducted behavioral tests at the same time. *Open field test:* Mice were placed in the center of an open-field chamber (Omnitech SuperFlex), and their movements and activities were observed and recorded for a period of 30 min under normal conditions. The data analysis was conducted based on total distance traveled. *Rotarod test:* All animals were pretrained for three consecutive days in order to reach a stable performance. On Day 1, mice were trained on the rotating rod at speed of 5 rpm in a period of 300 s three times. On Day 2 and Day 3, mice were trained on rod accelerating from 5 to 40 rpm within 60 s in a period of 300 s three times. The final test was performed on day 4. The time each mouse stayed on the rotating rod was

recorded, and the average duration from three repeated tests of each animal was used for data analysis.

Reference

1. Shi Y, et al. Mouse and human share conserved transcriptional programs for interneuron development. Science (New York, NY). 2021;374(6573):eabj6641.
2. Xu P, et al. Human midbrain dopaminergic neuronal differentiation markers predict cell therapy outcomes in a Parkinson's disease model. The Journal of clinical investigation. 2022;132(14).
3. Li XJ, et al. Coordination of sonic hedgehog and Wnt signaling determines ventral and dorsal telencephalic neuron types from human embryonic stem cells. Development (Cambridge, England). 2009;136(23):4055-63.
4. Wu M, et al. Transplanted deep-layer cortical neuroblasts integrate into host neural circuits and alleviate motor defects in hypoxic-ischemic encephalopathy injured mice. Stem cell research & therapy. 2024;15(1):422.
5. Ma L, et al. Human embryonic stem cell-derived GABA neurons correct locomotion deficits in quinolinic acid-lesioned mice. Cell stem cell. 2012;10(4):455-64.
6. Ji X, et al. Functional reconstruction of the basal ganglia neural circuit by human striatal neurons in hypoxic-ischaemic injured brain. Brain : a journal of neurology. 2023;146(2):612-28.
7. Nimitvilai S, et al. Dopamine D2 receptor desensitization by dopamine or

corticotropin releasing factor in ventral tegmental area neurons is associated with increased glutamate release. Neuropharmacology. 2014;82:28-40.
