# Supplementary figures and images for: 3D cultured human medium spiny neurons functionally integrate and rescue motor deficits in Huntington’s disease mice
Source: J Clin Invest. 2025 Oct 15;135(20):e187941. doi: 10.1172/JCI187941 (PMC12520685; doi:10.1172/JCI187941)

Full unedited blot/gel for Figure 10

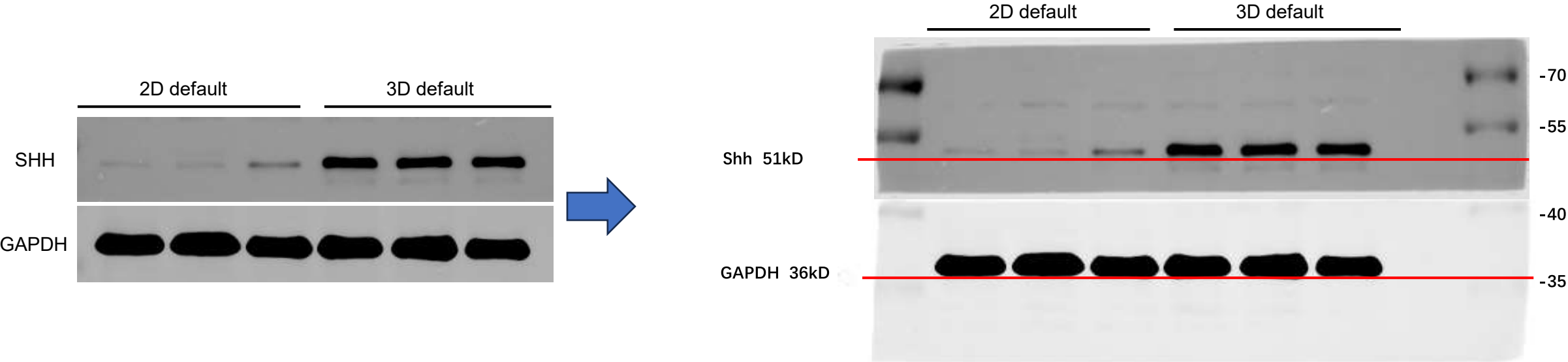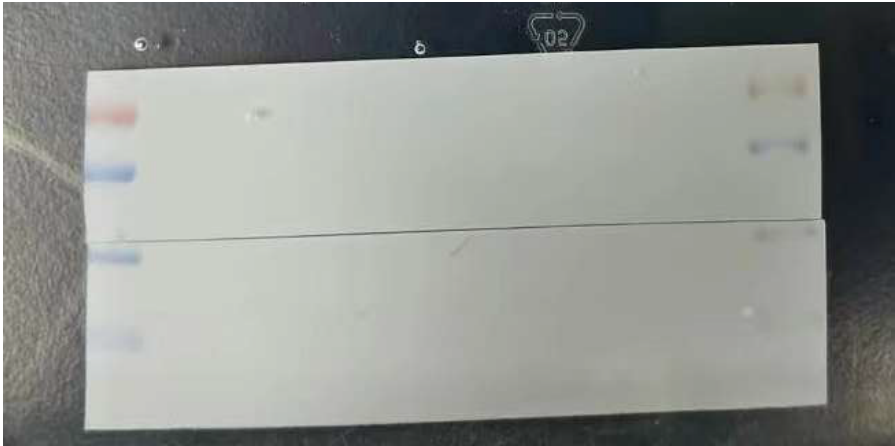

Supplement: Unedited blot and gel images [file jci-135-187941-s149.pdf]
